# Seasonal time trade-offs and nutrition outcomes for women in agriculture: Evidence from rural India

**DOI:** 10.1016/j.foodpol.2021.102074

**Published:** 2021-05

**Authors:** Vidya Vemireddy, Prabhu L. Pingali

**Affiliations:** a Indian Institute of Management Ahmedabad, India; b Charles H. Dyson School of Applied Economics & Management, Cornell University, United States; c Tata-Cornell Institute for Agriculture and Nutrition (TCI), Cornell University, United States

**Keywords:** Time use, Nutrition, Agriculture, Gender, India, Panel data, Opportunity cost

## Abstract

Women in agriculture are involved in agricultural activities and are solely responsible for household-level unpaid work. They face severe time trade-offs between agricultural and household activities across crop seasons. Recent literature suggests that these time trade-offs may negatively impact their nutrition. However, there is no quantitative evidence exploring this relationship within an agricultural context. This paper addresses this research gap by analyzing the relationship between women’s time trade-offs and their nutritional outcomes. Using a unique ten-month primary panel data of 960 women from India, our findings show that women are severely time-constrained, as they contribute significantly to agricultural as well as domestic work. Our results show that during peak seasons relative to lean seasons, women’s time trade-offs (rising opportunity cost of time) are negatively associated with the intake of calories, proteins, iron,zinc and Vitamin A. We show that this negative relationship is manifested severely among women who are landless and cultivate paddy alone (food crop) or paddy and cotton (mixed crop). This study highlights the gendered role of agricultural activities in rural households and the need to recognize time as a scarce resource when implementing policies and programs involving women in agriculture. We contribute to the literature of agriculture-nutrition linkages by examining the the time use pathway in detail. Besides providing novel metrics, we discuss several policy implications to reduce women’s time constraints and enhance their nutrition.

## Highlights

• Women in agriculture allocate significant time to agriculture and household activities.
• Rising time demands during peak season agriculture may affect women’s nutrition.
• Women’s time trade-offs negatively affect consumption of various nutrients.
• These associations vary by cropping pattern & land ownership.
• Policies must recognize women’s time constraints to improve their nutrition.

## 1 Introduction

In developing countries, women play a significant role in food production and nutrition provision by engaging in agriculture, processing, and food preparation within the household (Hyder et al., 2005).There is a growing recognition that women's agriculture involvement is increasing (ILO, 2016). Despite their significant involvement in agriculture, the burden of malnutrition is disproportionately more likely among women than men (FAO, 2016). Bringing equity in nutritional status requires an understanding of nuances in agriculture-nutrition linkages with a gender lens. However, relatively few studies explore the link between women's work in agriculture and their own nutritional outcomes.

Estimates from cross-country datasets show that women spend about 32 percent of their time on agricultural activities such as transplanting, weeding, harvesting (FAO, 2011). They are also solely responsible for unpaid activities in many contexts - like cooking, cleaning, collecting fuel, fetching water, and caring for children/family. For instance, women in India spend an average of 300 min in unpaid work on a typical workday and about nine times more in unpaid work than men (Charmes, 2015). Given the multiplicity of roles, women face constraints on their time due to competing demands between paid and unpaid work.

Recent literature notes that women's increasing role in agriculture may negatively impact nutritional outcomes because of time trade-offs with household food preparation (Johnston et al., 2018). Providing and ensuring a nutritious meal within a family is a time and resource-intensive process (Hyder et al., 2005). An increase in time spent on agriculture/income-generating activities translates into less time for household production activities. These time trade-offs can be measured through the opportunity cost of time, i.e., earnings forgone by a woman for every minute spent in unpaid activities, vis-a-vis paid work.

Further, these associations are likely to vary across agricultural seasons. Seasonality plays a crucial role in the division of tasks across gender and also time allocation. During peak seasons of agriculture relative to lean seasons, the increased demand for women's time (greater opportunity cost) can further affect nutritional outcomes. Yet, we find no studies that explore the role of women's time trade-offs in explaining their nutritional outcomes, such as diet quality across seasons.

This paper presents evidence on seasonal time trade-offs between agricultural and household activities for women and consequent nutrition outcomes. We hypothesize that during peak seasons, relative to lean seasons, women face severe time-constraints which are negatively associated with nutritional outcomes such as the consumption of calories, fats iron, protein, zinc and vitamin A. Our primary research objective is to investigate the relationship between women's seasonal time trade-offs and their nutritional outcomes. To do so, we interpret time trade-offs through the opportunity cost of time of women in agriculture. We use women's intake of calories, proteins, fats, iron, zinc, and vitamin A as nutritional outcome variables. For the first time, we collect individual-level nutrient consumption through an extensive recipe standardization exercise.

Further, we study how these time constraints imposed by seasonal agriculture associate with nutrition. Specifically, we hypothesize the increased time demands in agriculture affect the time spent for cooking and consequently nutrition outcomes. We examine how this relationship varies across land-ownership and cropping patterns. We examine this relationship using household panel data from 960 households from rural Maharashtra, India. The data from the sample households were collected once a month for a ten-month period from October 2016 to January 2018. All data used in the study was personally collected by the senior author on this paper.

### 1.1 Time constraints and women's nutritional outcomes

Linking women's participation in agriculture to nutrition outcomes has drawn much attention in recent literature. Herforth et al. (2012), note that smallholders and women, in particular, are more likely to be malnourished. While several pathways can enhance nutritional outcomes via agriculture (Ruel & Alderman, 2013), Kadiyala et al. (2014), note that agriculture involves gendered division of tasks that influence women's time for self-care and their children. Jabs & Devine (2006), emphasize that time constraints change the food consumption patterns - decrease food preparation at home or increase the consumption of fast foods/convenience foods. These changes in diets are less healthy and may contribute to obesity and other forms of malnutrition.

Among developing countries,^1^ time constraints have been modeled in terms of the opportunity cost of time. Results suggest that the opportunity cost of women's time is negatively related to household level nutrient intake, as in the case of rural South India (Behrman and Deolalikar, 1990, Pitt and Rosenzweig, 1985) and positively associated with the consumption of time-saving foods, such as processed bread in Sri Lanka (Senauer et al., 1986). However, limited studies explore the role of seasonality and use individual-level nutrient estimates of women. More recently, in a systematic review Johnston et al. (2018), emphasize women's time commitments in agriculture as laborers and farmers. While discussing the complexity and variation in household response to increased time burdens, they show evidence that women's time burden may increase due to agricultural interventions. The review highlights the need to understand the pathways through which time constraints affect nutritional outcomes. Using underweight and overweight relative to normal Body Mass Index (BMI) as outcome measures among women Padmaja et al. (2019) show that increased time on high-energy activities in agriculture is positively associated with underweight women. Though these studies jump to outcomes such as BMI and diet diversity, Kadiyala et al. (2014) note that more likely impacts are on immediate food consumption behavior. However, we find no studies that explore specific nutrient consumption such as calories, iron, zinc etc., consumed by women, which would ultimately have long-term implications on nutrition outcomes such as BMI. Besides the limited evidence presented above, the literature also groups women as one homogenous category and does not discuss the differences among cropping patterns, land size, and the underlying mechanisms that explain these relationships.

Our study makes the following key contributions. It’s the first study that associates seasonal time trade-offs with specific women's nutrition outcomes such as the consumption of calories, iron, etc. Among the several pathways through which agriculture can impact nutrition, there are limited studies that look at how time use as a pathway (Johnston et al., 2018). Through this study we contribute to the literature on agricultural- nutrition pathways by examining the time-use pathways in a detailed manner. We further this literature by looking at the variation across cropping patterns, income groups and provide first insights into the underlying mechanisms. These estimates also contribute to the knowledge of women’s opportunity cost of time in an agricultural setting and a developing country context. We measure multiple outcomes of women’s actual consumption of nutrients. In a novel attempt that is nuanced, precise, and context-specific, we standardize 502 locally consumed dishes in terms of nutrient composition and time taken for preparation. Methodologically we provide precise nutrient intake estimates at the individual level and provide a large novel dataset with individual-level diet and time use information that spans across seasons. Besides examining the time-use pathway, we provide detailed estimates of women and men's seasonal time allocation to agriculture and household activities. This adds to the extensive literature on the work and time burdens experienced by women in agriculture and the importance of recognizing these time trade-offs. This study's findings lend support for introducing labor-saving technologies to reduce women’s time burdens in agricultural and household activities and enhance their nutrition outcomes.

The rest of the paper is structured as follows. Section two provides details of the fieldwork and data collection process. Section three describes the measures and the method of analysis, followed by results in Section four. Finally, we conclude by discussing the policy implications in Section five.

## 2 Fieldwork and data collection

### 2.1 Fieldwork

**Fig. 1:**
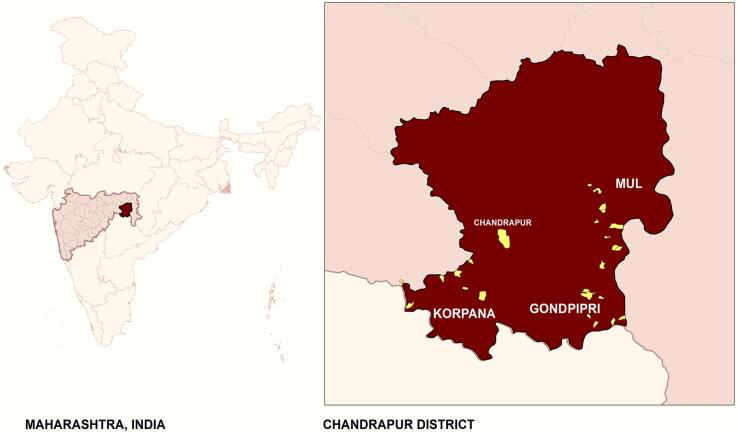
Site map.

**Fig. 2:**
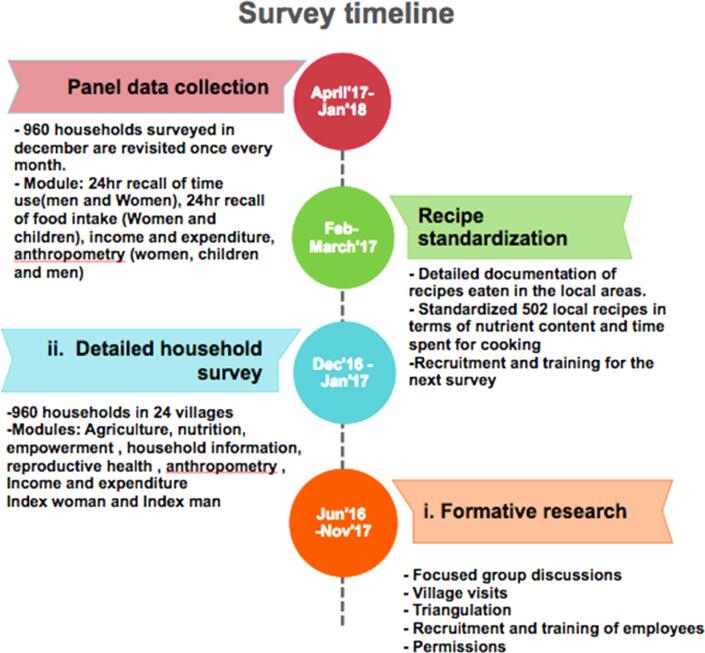
Survey timeline.

We administered three primary surveys for obtaining the data. These surveys were conducted from December 2016 to February 2018, and details of each survey, including the map, are given in Fig. 1, Fig. 2. We obtained ethical approval for this study from the Institutional Review Board at Cornell University. Oral consent for all the surveys was obtained and recorded in tablets as a part of computer based surveys.

#### 2.1.1 Context and sampling

This study was conducted in the rural areas of the Chandrapur district of Maharashtra, India. Chandrapur is in the state of Maharashtra and was selected because of its differences in cropping patterns. To the west of Chandrapur, cash crops such as cotton are cultivated and paddy in the east. According to 2011 Census of India, more than half of the population in Chandrapur is engaged in agriculture as a source of principal employment. This district is also characterized by poor nutritional status, particularly in rural areas. According to the National Family Health Survey 2014–2015, about 37.1 percent of women are below normal body mass index (BMI) levels, and about 50 percent of women are anemic (NFHS, 2015).

Three blocks were identified during the formative research: Mul, Korpana, and Gondpipri, based on the differences in the cropping patterns. Mul is in the paddy-growing region, Korpana is in the cotton-growing region, and the Gondpipri block has households that engage both in paddy and cotton cultivation. After triangulating the lists from block-level and village-level government institutions, household and village lists were generated. 24 villages were randomly selected based on probability proportional to the population size sampling method (8 villages per block). Three hundred twenty households per block (40 households per village) were selected, based on formative research in 2014, bringing the total sample size to 960 households. The sample selection process is described in detail in Gupta et al., 2017.

In each of the 960 households, a representative woman and man were interviewed, usually a husband and a wife. The most involved woman, who was responsible for the household and working in agriculture, was interviewed. If there were multiple women in the household, we selected the woman who is most engaged in agriculture and household activities. The questionnaires were piloted and were created after conducting focus group discussions and in-depth interviews across the district to understand the local cropping patterns and allied agricultural activities in which households engage. During June 2016 –February 2017, detailed focused group discussions around time allocation patterns in agriculture and dietary intake were conducted. We collected- i) high-frequency data on time use and diets; ii) wages and market-level prices^2^.

#### 2.1.2 Time use, diets, wages, and market-level prices

To obtain data on detailed time allocation patterns and diets of women across agricultural seasons, we conducted a ten-round (ten months) primary survey from April 2017 to January 2018. Each household was visited once every month on a randomly chosen day, except the weekends, and the same men and women were interviewed as the household survey explained above. Further, we also collect men's time use data for comparison. Since most households only crop in the Kharif^3^ season, we excluded the data collection for February and March 2018. The activities in February and March 2018 resemble patterns in April 2017.

In each visit, the respondents were asked to recall their time use and dietary intake in the past 24 h. The comprehensive list of all the activities was first cataloged using the recommendations from the Indian Time Use Study (Hirway, 1979, Hirway, 2009). These activity classifications were modified and adapted to the local context of Chandrapur district (see A1 ). Detailed 24-hour recalls of the diets were also collected for women and children. Further, income and expenditure data, health information, height, and weight (for women and children) data were collected on each visit (see A2).

We collected daily wages for women and men at the village level. Wages were collected for every village each month on the day of the survey. Similarly, we collect market prices of all foods from all the neighboring village-level markets, which were collected at each visit (each month) for ten months.

#### 2.1.3 Recipe standardization

This research involved standardizing recipes of locally consumed dishes, to obtain precise measures of nutrients and the time taken to prepare and cook these recipes. India's nutrient information is compiled by the National Institute of Nutrition in India (NIN) and captures nutrients available in edible portions of raw foods. Two major issues arise for measuring the nutrient intakes per day across seasons: (1) it is difficult to obtain precise estimates of home-prepared meals; and (2) the time-intensive nature of 24-hour dietary recall methods increase the respondent burden significantly (Coates et al., 2012). Considering that most households in rural India consume home-prepared meals, it was necessary to arrive at the recipe level estimates for both macronutrients and micronutrients. For accurate measurement of nutrient intakes and to reduce respondent burden, we conducted recipe standardization of all the most commonly eaten dishes. Following the methodology adopted by Harvest Plus, we first conducted detailed focused group discussions to develop a catalog of most of the recipes eaten in the region (Gwatkin et al., 2007). After cataloging, a detailed record of the process, time spent in preparation, cooking, weights of each ingredient used, and cooking method were recorded while standardizing the process. However, for the first time ever, the time taken to prepare these meals was also standardized along with the nutrients. Both - the time taken for preparing the ingredients and the actual cooking time were recorded.

Detailed nutrition data was compiled using the standardized recipes and nutrient information from the food composition tables from India (Longvah et al., 2017). For each standardized local recipe, a data set was created that contains information on calories (kcal), macronutrients (proteins, fats, carbohydrates), micronutrients, and cooking time. The catalog of the foods eaten was used to develop a codebook (in English and Marathi -the local language), which could be used as a tool in the multi-round survey for collecting 24-hour dietary intake data. The respondent burden was minimized by recalling recipes and associated quantities, using standardized cup and spoon measures, instead of recalling ingredients in each recipe (see supplementary material for the recipe names)^4^.

## 3 Methods

For this study, we use data from all the primary surveys mentioned in the previous section.

### 3.1 Measuring nutrient intakes

As a part of the 10-month panel study, women were asked to recall recipes eaten in the past 24 h on a random day during each month of the study. They were also asked to recall the quantities consumed using the standardized measures of cups and spoons. Using the standardized recipe-level nutrient information, we calculate individual-level daily nutrient intakes for women. Nutrient consumption per day was measured in terms of: (1) calories (kcal); (2) macronutrients: protein (g), fats(g); and (3) micronutrients: iron (mg), zinc (mg), vitamin A (µG). Calories typically reflect individual's energy intake and are extremely important in determining the individual's overall health. Similarly, proteins and fats to are essential, as they serve the purpose of muscle development and providing the required energy for the body. We exclude carbohydrates in this analysis, because the calorie measures capture the impact of carbohydrates.

### 3.2 Measuring time trade-offs- the opportunity cost of time

In this paper, we measure time trade-offs via women's opportunity cost of time, that is, the wages forgone when a woman is engaged in household activities instead of working for the market wage. We use village-level women's wage rates to measure the opportunity cost of women's time. The use of opportunity cost of time in lieu of time use is common in the economics literature (Prochaska and Schrimper, 1973, Senauer et al., 1986). Studies note that time use can impact nutrition/health indicators and vice-versa- thereby inducing simultaneity bias (Behrman and Deolalikar, 1990, Bhargava, 1997). This has been noted both in theoretical modelling as well as empirical work. Therefore, we use exogenously determined local level market-level wages to value the time trade-offs and minimize the bias. To examine the workloads across seasons and the underlying mechanisms, we use the time use data collected in our survey. The time use variables collected in this survey are detailed in A2.

Further, our rationale for using agricultural wages as an opportunity cost to estimate the relationship across the sample is as follows: In our study, women and men are actively engaged in farming. Findings from the survey suggest that about 85% of women in the sample participate in wage work. This is further supported by the fact that the average size of landholding is 3 acres (1.2 ha). About 31 percent of them are landless, while most of the sample households own below 5 acres of land. They participate in farming either through their own land, leased land and/or as agricultural labor. Given the large percentage of women who participate in farming activities as agricultural labor, the village level market wages serve as the minimum value for measuring time trade-offs. For women who participate in work on their own farms as well, we argue that the local wage will serve as the floor opportunity cost. However, we recognize that this does not estimate the true opportunity cost. In the household survey, about 15 percent of the women do say that they engage in secondary and tertiary sectors besides agriculture. However, when we look at the time use patterns, in addition to agriculture involvement, we find that only about 23 households spend their time in secondary and tertiary activities across the ten months. In terms of their time spent, an average across all the ten months does not exceed 5 min among these 15 households, and the maximum being 46 min during November 2017 (see A3 & A4). For these reasons, we believe that using agricultural wages is the most suited method. We also show a specification where we drop these households (see A5).

### 3.3 Estimation

The reduced form theoretical model shows nutrient intakes as a function of wages, market prices, and non-labor income^5^. Following the theoretical model, we estimate the relationship between the opportunity cost of time and nutrition by the following individual fixed effects model:

[1] $$N_{\mathrm{ihvt}}^{j}=\beta_{0}^{j}+\beta_{1}^{j}{\mathrm{Wage}}_{\mathrm{vt}}^{f}+\beta_{2}^{j}P_{\mathrm{vt}}+\beta_{3}^{j}Z_{\mathrm{ihvt}}+\beta_{3}^{j}H_{\mathrm{hvt}}+{∊}_{\mathrm{ihvt}}^{j}$$

Where *i* refers to a woman, *j* is a specific nutrient, *h* is the household, *v* is the village, *t* is the season in which the women were interviewed. $N_{\mathrm{ihvt}}^{j}$ includes a vector of nutrition indicators: minimum dietary diversity of women, calories (kcal), protein (g), fats(g), iron (mg), zinc (mg), vitamin A (µG).In this study, we are interested in analyzing the role of the opportunity cost of time on the nutrient intake of women. In other words, as seasons change, we are interested in the association between the changing opportunity cost (value of time) on changes in woman's nutrient intake. There is enough variability in daily nutrient intake, which is affected by both time-varying factors and time-invariant factors. There is a high probability of omitted variable bias in cross-sectional settings, which produces biased and inconsistent coefficients. Panel data controls for the effect of all the fixed factors together that can impact the outcomes. However, time-varying factors still need to be controlled. The individual fixed effects control for all the genetically related factors that may impact nutrient intakes, such as metabolic rates, tolerance to diseases, and immunity. The household-level factors, such as housing conditions, sanitation, water facilities, and household health and nutrition, are also time-invariant and are controlled for in the individual fixed-effects model, as they are likely to be correlated with incomes.

In the specification shown above, ${\mathrm{Wage}}_{\mathrm{vt}}^{f}$ refers to village-level wages for women, which were collected for every village each month on the day of the survey. These wages signify the opportunity cost of women's time. With an increase in the opportunity cost of time (${\mathrm{Wage}}_{\mathrm{vt}}^{f}$), the price of time spent at home increases. Theoretically, the increase in the marginal cost of producing nutrients at home has two effects: an increase in demand for agricultural work due to increased wages. This demand, in turn, reduces time spent on cooking but also increases the amount that can be spent on purchasing raw or processed foods. Alternatively, as time spent on cooking decreases, the number of nutrients available may decrease. If the income effect of the wage increase dominates the substitution effect, we will see an increase in nutrition indicators through an increase in both time spent in agriculture and time spent in nutrition-enhancing activities. On the other hand, if the substitution effect dominates the income effect, a rise in wages leads to a decrease in nutrition through an increase in time spent in agriculture (corresponding to decreased time spent in nutrition-enhancing time).

Availability of food impacts nutrition, as women purchase these food items, such as raw ingredients to prepare meals. In this context, from our field knowledge, we observed that women often buy their weekly groceries from a local market nearby their respective villages. To control for the impact of availability, we control for $P_{\mathrm{vt}},$which represents the vector of prices of raw food items purchased from a nearby market. $Z_{\mathrm{ihvt}}$refers to individual-level factors that are time-varying, such as whether the individual is sick or not on the interview day. $H_{\mathrm{hvt}}$ includes all the household-level factors that are time-varying, such as non-food expenditures or whether an individual is sick in the household. Seasonality plays a critical role in nutrition, as variations in the climate such as rainfall and temperature can impact the consumption of nutrients and the amount of time spent in agriculture. Women's tasks vary with the seasons, impacting their time spent in agriculture, and their involvement in nutrition-enhancing activities may be affected due to this variation. To our knowledge, this is the first study that empirically controls for seasonality in this framework to analyze the impacts across seasons. We construct a season variable by clustering the months, based on major agricultural activity periods: land preparation (April–May), sowing (June–July), transplanting (August–September), and harvesting (October–November) and (December to January).

## 4 Results

### 4.1 Sample characteristics

Chandrapur district has a population of 2.2 million, according to the Census of India, 2011. Table 1 shows the summary statistics of the sample used for this study. Initially, 960 households were sampled, but six households dropped out during the ten months due to death or permanent migration among these families, reducing the total to 954 households. The average size of the families is about four, reflecting a more nuclear nature of families. The average age of women is around 37 years, and about 47 percent of the sample has no education or primary education. The average number of children under the age of 18 is 1.14 children per household.

**Table 1:** Summary statistics (full sample).

Variable (N = 954)	Mean	Min	Max
Ownership of land (acres)	3.04	0	35
No. of children (0–6 years)	0.34	0	3
No. of children (under 18 years)	1.14	0	4
Household size	4.42	0	9
Access to irrigation	0.24	0	1
Access to electricity	0.91	0	1
Women’s age	36.96	21	50
**Women's Education (%)**			
No education	33.6%		
Primary	13.9%		
Secondary	31.9%		
Higher secondary and above	20.5%		

This table reflects the characteristics of the sample that is used in the analysis.

Most of these households are agrarian and marginal—smallholder farmers with an average landholding size of 3 acres. Twenty-four percent of households have access to irrigation. Otherwise, agriculture is mainly rainfed in this region. Agricultural activities in agriculture are divided between men and women in many developing countries, and similar patterns are observed in Chandrapur as well (Rao and Raju, 2020). In cotton-growing regions, women engage in activities such as cotton picking, weeding, fertilizer application, and land preparation activities. In paddy-growing regions, weeding, transplanting, harvesting, and postharvest activities are typically the work of women. Table 2 shows the activities that women engage in across seasons for both paddy and cotton farming.

**Table 2:** Agricultural activities across seasons (women).

Month	Cotton	Paddy
June	Sowing	Land preparation /transplanting
July	Weeding and fertilizer application	Transplanting
August	Weeding and fertilizer application	Transplanting
September	Land preparation and fertilizer application	Weeding
October	Cotton picking and fertilizer application	Weeding
November	Cotton picking	Harvesting
December	Cotton picking	Harvesting/Storage/Processing
January	Cotton picking	Harvesting/Storage/Processing
February	Relatively free months	Other activities
March	MGNREGA and other activities	Other activities
April	Soil cultivation, banking arrangements and other off-farm work	Other activities
May	Soil cultivation, banking arrangements and other off-farm work	Other activities

Fig. 3a shows the distribution of time spent in agriculture, cooking, and domestic work. As shown in these figures, the average time spent in agriculture reaches up to 333 min in the peak seasons of sowing (July–August) and harvesting (October–November). Due to small landholdings, most women engage in agricultural wage work even if they own land, and the wage rates are market-level wages.

**Fig. 3a:**
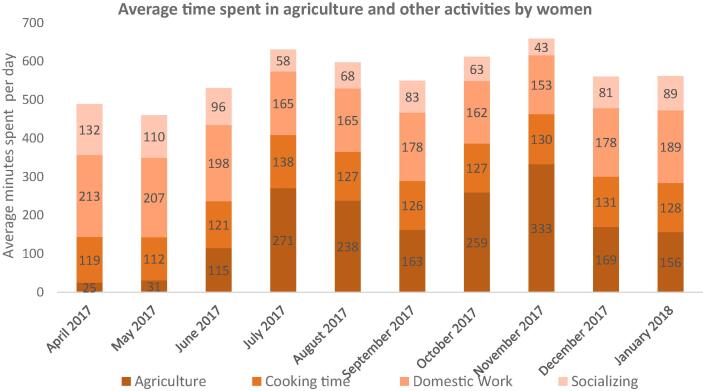
Time spent in agriculture, domestic work, cooking and socializing by women across rounds.

We analyze men's time use, as shown in the second panel of Fig. 3b. Men spend almost equal time when it comes to time spent in agricultural work. However, if we compare socializing time, men spend about three times as much as women in social activities, averaging 292 min across the seasons. Their contribution to domestic work and food preparation is quite low, averaging about 33 min and 39 min, respectively. This reflects that men do not face time constraints, as do women, as they are not involved in household-related activities. Therefore, we argue that women's wages truly reflect this time trade-off between agriculture and time for home-based activities, while men's wages reflect a pure income effect.

**Fig. 3b:**
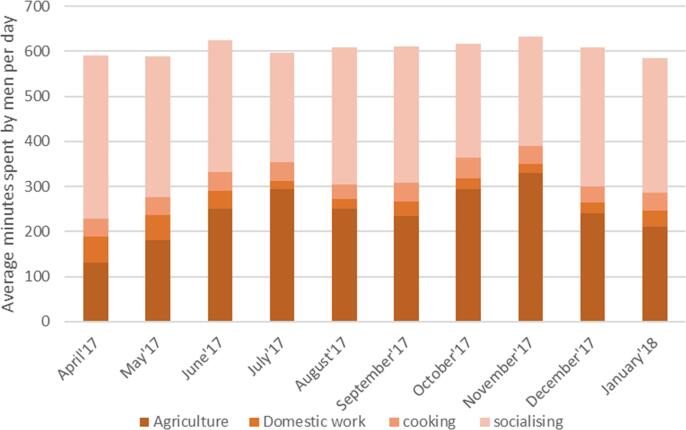
Time spent in agriculture, domestic work, cooking, and socializing by men across rounds.

There is a variation in nutrient intakes across seasons as well. The Indian Council for Medical Research (ICMR) reports the recommended daily allowance (RDA) for vital nutrients in Indian diets, and these are shown in Table 3. These RDAs are defined for men and women separately, based on the level of physical activity they engage in. We compare our average daily intakes for women across seasons with the RDAs in Table 3. It is important to note that, except for calories and fats, all other nutrients fall short of the RDAs. Particularly concerning are levels observed for proteins, iron, zinc, and vitamin A.

**Table 3:** Average intake of nutrients per day for women across regions and season.

	Calories (kcal)	Protein (g)	Fats (g)	Iron (mg)	Zinc (mg)	Vitamin A (µg)
Recommended dietary allowance per day (RDA)**	2,850	55	20	21	10	2,400
April–May	2,395.9	18.9	19.8	5.3	3.0	1,050
June–July	3,103.6	19.1	20.3	5.6	3.2	1,172
August–September	3,009.7	18.4	17.3	5.6	3.2	1,115
October–November	3,308.0	19.9	21.6	5.8	3.2	1,303
December–January	3,251.7	19.1	24.2	5.8	3.1	1,556.5

**Nutrient Requirements and Recommended Dietary Allowances for Indians, Indian Council of Medical Research, 2010

### 4.2 Women's opportunity cost of time and nutrition: Full sample

Table 4 explores the relationship between the opportunity cost of women's time and nutrition indicators. Panel A shows the effects without any controls, and Panel B has included control variables, such as market prices of foods, etc. Panel C includes both controls and season dummies. The individual-level fixed effects estimates show that an increase in wages is negatively associated with the intake of calories, protein, fats, iron, zinc, and vitamin A. All the regression models across Panels A, B, and C contains individual fixed effects. The associations are statistically significant after controlling for all the time-invariant factors for women, communities, and villages.

**Table 4:** Effect of opportunity cost of time on daily nutrient intakes of women per day (N = 8,002).

	Calories	(2) Protein	(3) Fats	(4) Iron	(5) Zinc	(6) Vitamin A	(7) MDDW^1^
	(kcal)	(g)	(g)	(mg)	(mg)	(µg)	
	b/se	b/se	b/se	b/se	b/se	b/se	b/se
**Panel A: No controls**							
Female wages (Rs./day)	0.014	–0.008***	0.004	–0.002*	–0.002**	0.573*	–0.001**
	(0.237)	(0.002)	(0.004)	(0.001)	(0.000)	(0.278)	(0.000)
Male wages (Rs./day)	2.269***	0.012***	0.017***	0.003**	0.002***	1.767***	–0.001***
	(0.220)	(0.002)	(0.004)	(0.001)	(0.000)	(0.293)	(0.000)
**Panel B: Controls**							
Female wages (Rs./day)	–0.745**	–0.013***	–0.003	–0.006***	–0.004**	–0.364	–0.000
	(0.285)	(0.003)	(0.005)	(0.002)	(0.001)	(0.351)	(0.000)
Male wages (Rs./day)	1.562***	0.010***	0.009*	0.003**	0.002**	1.501***	–0.002***
	(0.244)	(0.002)	(0.004)	(0.001)	(0.001)	(0.320)	(0.000)
**Panel C: Controls and Season dummies**							
Female wages (Rs./day)	–1.237***	–0.015***	–0.018***	–0.007***	–0.004***	–1.372***	–0.000
	(0.299)	(0.003)	(0.005)	(0.002)	(0.001)	(0.385)	(0.000)
Male wages (Rs./day)	0.787**	0.008**	0.000	0.000	0.001	0.556	–0.001***
	(0.281)	(0.003)	(0.005)	(0.001)	(0.001)	(0.371)	(0.000)

*Note*: All regressions use individual fixed effect models. Women who have not participated in agriculture at all across 10 months are excluded from the sample. Standard errors are clustered at the household level. Significance levels: + 0.10, * 0.05, ** 0.01, *** 0.001.

The control variables include daily market prices of rice, wheat, onions, potatoes, spinach, tomatoes and pigeon pea. a dummy variable for sickness of children, and a dummy for sickness of others in the household. Season dummies control for seasons and type of day as well.

1 Minimum Dietary Diversity Score for women (MDDW)

The agricultural wages measure the trade-offs women face with respect to their time spent in agriculture and non-agricultural activities. The regression results show that a 100-rupee increase in a woman's agricultural wages (opportunity cost of time) per day is associated with a decline in her calories equal to 112.3 kcal, 0.7 mg iron, 0.4 mg zinc, and 1.5 g protein. These associations suggest that during peak seasons relative to lean seasons, when agricultural activities demand more time from women, their cost of time increases for every minute spent on unpaid activities. Since we include women who have engaged in agricultural activities, the market-determined village-level female wages represent their least cost of time^6^. Since the nutrient intakes are reported as a 24-hour recall once monthly, we control for fluctuations of seasons and a type of day by including time and day dummies. We also control for price fluctuations in commonly eaten foods. An increase in staples prices, such as rice and wheat, leads to a significant increase in protein intake, fat, and vitamin A, suggesting that women substitute cereals with nonstaple foods. An increase in spinach's price, holding other variables constant, is associated with a significant decrease in all macro-and micronutrient intake, except calorie consumption. The sickness of an adult or a child does not seem to affect the intakes of women. It is important to note that male wages do not impact micronutrients after controlling for seasonality, suggesting that the time constraints are more important for women's nutrition than income constraints once we account for availability and seasonality factors.

### 4.3 Women's opportunity cost of time and nutrition: Subsample analysis

The size of the land owned is likely to impact time constraints for women. For instance, for the women who are landless or hold marginal land, spending time in agriculture may not be a choice due to a loss in earnings. The same may not hold for women who belong to households with large landholdings as they could hire labor. Gupta et al. (2017), show that 'women's empowerment levels varied significantly across cropping patterns and were highest among cash-crop growing households. Each crop has different agriculture activities across seasons, the time involved differs across crops. Subsequently, time constraints may behave in a different way across cropping systems. We also conduct various subsample analyses to see how land ownership and cropping patterns affect this relationship.

#### 4.3.1 Cropping patterns

The opportunity cost may have varying effects on the nutrient intakes based on the household's cropping pattern. As we can see from Table 5, the effects vary based on the type of cropping system that the women work in. In the food crop system (paddy-producing region), we see that the time constraint is binding. For all the nutrients, the association is negative and statistically significant. Whereas in the cash crop region, we see a positive association with specific nutrients and a negative for fat and vitamin A, but none of these are statistically significant. In the mixed crop region, too, we see negative associations, suggesting the more time/labor-intensive nature of the crop production process.

**Table 5:** Effect of opportunity cost of time on daily nutrient intakes of women by cropping pattern.

	Calories	(2) Protein	(3) Fats	(4) Iron	(5) Zinc	(6) Vitamin A	(7) MDDW
	(kcal)	(g)	(g)	(mg)	(mg)	(µG)	
	b/se	b/se	b/se	b/se	b/se	b/se	b/se
*Panel A: Cotton growing households (N = 3,014)*							
Female wages(Rs./day)	0.076	–0.001	–0.012	0.001	0.001	–1.334*	–0.000
	(0.593)	(0.005)	(0.010)	(0.002)	(0.001)	(0.611)	(0.001)
Male wages(Rs./day)	–3.718	–0.075+	0.077	–0.031	–0.009	6.649	–0.005
	(4.470)	(0.043)	(0.070)	(0.020)	(0.009)	(4.273)	(0.004)
*Panel A: Paddy growing households (N = 2,664)*							
Female wages(Rs./day)	–17.510**	–0.088	–0.067	–0.101**	–0.041*	–50.620**	0.011
	(6.722)	(0.068)	(0.125)	(0.033)	(0.017)	(16.346)	(0.007)
Male wages(Rs./day)	13.753***	0.061*	0.076	0.046**	0.020**	21.102**	–0.004
	(2.747)	(0.031)	(0.051)	(0.014)	(0.007)	(6.893)	(0.003)
*Panel A: Cotton and Paddy growing households (N = 2,324)*							
Female wages (Rs. /day)	–4.490***	–0.030**	–0.004	–0.017**	–0.007***	–6.120*	–0.000
	(1.037)	(0.011)	(0.017)	(0.006)	(0.002)	(2.393)	(0.001)
Male wages (Rs. /day)	–0.103	0.007	0.002	0.007	0.010	1.202	–0.003**
	(08.14)	(0.012)	(0.014)	(0.006)	(0.007)	(1.081)	(0.001)

*Notes*: 1. All regressions use individual fixed effect models. Women who have not participated in agriculture at all across 10 months are excluded from the sample. Standard errors are clustered at the household level. Significance levels: + 0.10, * 0.05, ** 0.01, *** 0.001. 2. The control variables include daily market prices of rice, wheat, onions, potatoes, spinach, tomatoes and pigeon pea, a dummy variable for sickness of children, a dummy for sickness of others in the household, and season dummies and type of day dummies.

#### 4.3.2 Land ownership

We can see whether time constraint is more or less binding based on land-ownership. We use the mean landholding size to categorize the sample and conduct a similar analysis. As reflected in Table 6, we find that the association is more significant and more statistically robust in households with smaller landholdings than women in households with larger land sizes. These results show that larger land sizes, which reflect higher incomes, play a role in mitigating the adverse time effects on nutrition.

**Table 6:** Effect of opportunity cost of time on daily nutrient intakes of women by land-ownership.

	(1) Calories	(2) Protein	(3) Fats	(4) Iron	(5) Zinc	(6) Vitamin A	(7) MDDW
	(kcal)	(g)	(g)	(mg)	(mg)	(µG)	
	b/se	b/se	b/se	b/se	b/se	b/se	b/se
*Panel A: ≤ 3 acres (N = 5,141)*							
Female wages (Rs./day) wages	–1.339	–0.017***	–0.021***	–0.008***	–0.005**	–1.753***	–0.000
	(0.381)	(0.004)	(0.006)	(0.002)	(0.002)	(0.526)	(0.001)
Male wages (Rs./day)	1.145***	0.009**	0.003	–0.001	0.001	0.569	–0.001
	(0.333)	(0.003)	(0.006)	(0.002)	(0.001)	(0.455)	(0.000)
*Panel B: greater than 3 acres (N = 2,861)*							
Female wages(Rs./day)	–1.056*	–0.012*	–0.012	–0.005*	–0.003+	–0.931+	–0.001
	(0.500)	(0.006)	(0.008)	(0.003)	(0.002)	(0.552)	(0.000)
Male wages(Rs./day)	0.106	0.004	–0.007	0.001	0.001	0.775	–0.002**
	(0.587)	(0.005)	(0.010)	(0.002)	(0.001)	(0.730)	(0.001)

*Notes*: 1. All regressions use individual fixed effect models. Women who have not participated in agriculture at all across 10 months are excluded from the sample. Standard errors are clustered at the household level. Significance levels: + 0.10, * 0.05, ** 0.01, *** 0.001. 2. The control variables include daily market prices of rice, wheat, onions, potatoes, spinach, tomatoes and pigeon pea, a dummy variable for sickness of children and dummy for sickness of others in the household, and season dummies and type of day dummies.

### 4.4 Mechanisms through which opportunity cost of time impacts women's nutrition

**Fig. 4:**
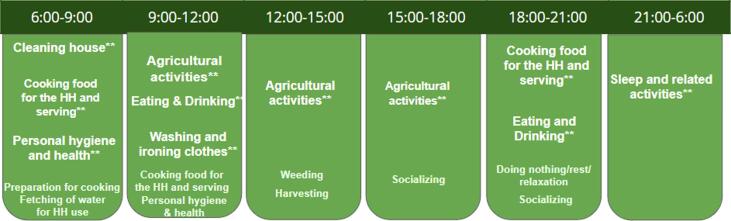
A schematic representing time spent by women throughout a typical day (** represents the major tasks reported by women in terms of time).

To understand the mechanisms through which the opportunity cost of time affects women's nutrition, we conduct several analyses based on time spent on agriculture. We hypothesize that increased time constraints reduce cooking time during peak seasons, subsequently playing a role in nutrition outcomes. In Fig. 4, we map the activities conducted by women throughout a typical day based on the time use survey responses. Agricultural activities are carried out during the mornings till the evening. Women cook meals twice a day, once in the morning and once in the evening. The increased time in agriculture can reduce the time for cooking during morning hours or evening hours. Based on the time of the day, factors such as lack of time, exhaustion can affect the meal preparation by women. They may choose to reduce cooking time, make dishes that are easy, less time consuming and require less effort. This in turn can affect nutrients derived from these meals

For analyzing, we categorize time spent in meal preparation in two-time slots, based on their meal preparation behavior (table 7). We look at the relationship between time spent in agriculture on cooking time in the morning and evening separately, and we find that for every ten additional minutes spent in agricultural work, cooking time is reduced by 4 min during the evening meal, and this result is robust to all specifications.

**Table 7:** Relationship between time spent in agriculture and time spent in cooking disaggregated by major meals within a day (morning and evening).

	(1)	(2)	(3)	(4)
				All controls
	b/se	b/se	b/se	b/se
	*morning*			
Time spent in agricultural activities (min)	0.009***	0.000	0.001	0.002
	(0.002)	(0.002)	(0.002)	(0.002)
	*evening*			
Time spent in agricultural activities (min)	–0.000	–0.004**	–0.003**	–0.003*
	(0.001)	(0.001)	(0.001)	(0.001)

*Notes*: 1. All regressions use individual fixed-effect models. Women who have not participated in agriculture at all across 10 months are excluded from the sample. Standard errors are clustered at at the household level. Significance levels: + 0.10, * 0.05, ** 0.01, *** 0.001. 2. Coefficients within each panel represent the specific outcome variable, that is, time spent on the activities. Specification (1) is without controls; (2) includes season and type of day dummies; (3) includes male wages, season, and type-of-day dummies; (4) includes all the variables in (3) plus market prices of rice, wheat, onions, potatoes, spinach, tomatoes and pigeon pea, and sickness of adults and children.

As shown in table 8, we further explore the direct link of agricultural time on the consumption of nutrients in the evening; we find that an increase in agricultural time reduces all nutrient intakes during the evening meal consumption. These results point to a reduction in cooking time due to increased time demands from agriculture during the evening meal preparation, with a subsequent decline in nutrient intake.

**Table 8:** Relationship between time spent in agriculture and nutrients disaggregated by major meals within a day (morning and evening).

	Calories	(2) Protein	(3) Fats	(4) Iron	(5) Zinc	(6) Vitamin A
	b/se	b/se	b/se	b/se	b/se	b/se
*Morning meal*						
Time spent in agricultural activities (min)	–0.049	–0.001*	–0.002***	–0.000	–0.000	–0.064
	(0.032)	(0.000)	(0.001)	(0.000)	(0.000)	(0.049)
*Evening meal*						
Time spent in agricultural	–0.123***	–0.002***	–0.004***	−0.001***	–0.000	–0.022
activities (min)	(0.036)	(0.000)	(0.001)	(0.000)	(0.000)	(0.058)
N	8,002	8,002	8,002	8,002	8,002	8,002

*Notes*: 1. All regressions use individual fixed-effect models. Women who have not participated in agriculture at all across 10 months are excluded from the sample. Standard errors are clustered at the household level. Significance levels: + 0.10, * 0.05, ** 0.01, *** 0.001. 2. Coefficients within each panel represent the specific outcome variable, that is, time spent on the activities. Specification (1) is without controls; (2) includes season and type-of-day dummies; (3) includes male wages, season, and type-of-day dummies; (4) includes all the variables in (3) plus market prices of rice, wheat, onions, potatoes, spinach, tomatoes and pigeon pea, and sickness of adults and children.

## 5 Conclusion and discussion

Using novel primary panel data and methods, we examine the relationship between women's opportunity cost of time and their nutrient intake. We hypothesize the following: as seasons change, the pressures on women's time in agriculture change. During peak seasons relative to lean seasons, they experience time constraints that influence their household activities. These different time pressures induce a trade-off for women's time for nutrition-enhancing activities, such as cooking and domestic activities, which may have adverse consequences for nutrition. In our analysis, we find that working more extended hours in agriculture, or in other words, the rising opportunity cost of women's time, is associated with a reduction in nutrient intake in terms of calories, proteins, fats, iron, and zinc.

Using first of its kind, detailed 10-month time use data for both women and men across seasons, we first show that women disproportionately bear the work burdens, as they work in agriculture and are solely involved in domestic work, food preparation, and care-related activities. Our analysis shows this distinction very categorically—on average, a woman spends almost the same time as men in agriculture, but men spend limited time in food preparation, domestic work, and care activities. In contrast, women spend more than 300 min daily on cooking and other domestic work-related activities, such as cleaning the house, washing utensils, clothes, etc. In peak seasons of sowing, transplanting, and harvesting, we show that women's extra hours in agriculture translate to reduced food preparation time. These findings suggest that women's engagement in agriculture is high and similar to men's. Yet, there is no subsequent reduction in the domestic activities for women during peak seasons. Time pressures in agriculture are also associated with less time for sleep, and rest-related activities, impacting women's overall health. Our findings expand the knowledge on women in agriculture by examining variation in time use and nutrition outcomes across cropping patterns, seasons, and land-ownership.

Our results also contribute to the literature by quantifying specific nutrient intakes. After controlling for all the factors, we find that increased time constraints for women across seasons as reflected by the opportunity cost of time translate to reducing their intake of calories, proteins, fats, iron, zinc, and vitamin A. These results hold even after the inclusion of the income of male members of the household, suggesting that time constraints are binding even after controlling for income. We analyze the effects of income-rich households by conducting a subsample analysis using land ownership. We see that time constraints are most binding on women who are landless and almost insignificant when it comes to women who own land. We split the sample by their cropping pattern—cash crops, food crops, and mixed crop households to analyze agriculture pathways. Here, we find that paddy-growing and mixed crop-growing households have pronounced negative impacts of rising time constraints on their nutrient intakes, while cotton-growing households do not have the same experience.

Our field experiences suggest that women think in terms of recipes and not food groups, and they also tend to misjudge the time it takes to cook a recipe. One can compare time savings in food preparation between ready-to-eat food and home-cooked food. However, the existing literature stops short when it comes to food prepared at home. We fill these research gaps by methodologically contributing to the existing literature; we conceptualized and standardized 502 recipes that are locally consumed—thus, considering the local context, tastes, and preferences—in terms of the time taken to prepare and cook a recipe and the weights of each ingredient used in every recipe. We find that this method is beneficial for the collection of panel data.

Our results have several policy implications. Firstly, several strategies to reduce women's time and work burdens by promoting labor-saving technologies for women are extremely important. In a rural context, agriculture and domestic work activities are not only time-consuming but are also arduous. In such a scenario, it is vital to introduce labor-saving strategies both in agriculture as well as in domestic work. Time-saving/ labor-saving technologies and assets in agriculture or at home can be beneficial strategies, as suggested by Johnston et al. (2018). These technologies should be context-specific, account for the production environment, and target the appropriate population. Secondly, women's wages are significantly lower, and there is enough evidence to suggest that improved incomes of women lead to the household's better well-being. Increasing incomes and strategies to promote women's empowerment (Sraboni et al., 2014) through enhanced decision-making powers and control over income are imperative. Thirdly, our findings confirm that women contribute to agriculture significantly as farm laborers, farm managers in various activities spread across seasons. This would mean the policy should be aligned to women's needs in agriculture- be it technology, finance, and extension. Finally, our results clearly show that designing agricultural interventions and development programs must recognize the consequences of increased time burdens and the adverse effects on nutrition. The programs should make sure that the benefits of participation in agriculture outweigh losses such as time for household activities and leisure.

Even if time burdens are managed, enhancing women’s nutrition would require considerable effort to ensure the consumption of diverse diets throughout the year. This requires a reorientation of Indian public policy in several ways. On the supply side, this requires moving towards nutrition-sensitive food systems from largely staple-centric production systems (Pingali, 2015). This can be further supported by making provisions of non-cereal foods through the public distribution system. Community awareness through behavior change communication programs for promoting nutrient-rich foods are needed on the demand side. Further, to make sure nutrient-dense foods are available and affordable, enhancing market infrastructure and food availability across seasons is essential.

## CRediT authorship contribution statement

**Vidya Vemireddy:** Investigation, Data curation, Project administration, Conceptualization, Formal analysis, Methodology, Writing - original draft. **Prabhu L. Pingali:** Conceptualization, Funding acquisition, Validation, Writing - review & editing.

## Declaration of Competing Interest

The authors declared that there is no conflict of interest.

## Footnotes

1 In a nonagricultural context, literature among high-income countries shows that a rise in the opportunity cost of women’s time increases consumption of food away from home (Davis, 2014; Gawn et al., 1993; Prochaska & Schrimper, 1973; Cawley & Liu, 2012). For instance, Cawley and Liu 2012, show that maternal employment is associated with 17 fewer minutes cooking and 37 fewer minutes caring for children. They suggest that these findings suggest plausible mechanisms liking maternal employment and childhood obesity.

2 In addition to time use, diets and wages information we use household level information. This household information is a panel where the information was collected through two surveys- one is 2014 (detailed in Gupta et al., 2017) and the other one in 2016 (see A1). In this study, we use the household information for the descriptive of households and sub-sample analysis.

3 Kharif crops, are the crops which are grown during the monsoon or rainy season. There are two main cropping cycles in India – Kharif and Rabi (winter crop).

4 The supplementary material is in local language (Marathi).

5 $N=N\left(w,p_{r},p_{p},p_{x},V;Z\right)$

6 We exclude women who engage in other primary activities besides agriculture other specificaions. However, they could be engaged in more activities besides agriculture during the day from a secondary activity classification. In that case, the magnitude of the estimates are likely to be more since the opportunity cost of time is going to be more (appendix A4,A5).

Appendix A and B Supplementary data to this article can be found online at https://doi.org/10.1016/j.foodpol.2021.102074.
