# Supplementary material for: Seasonal time trade-offs and nutrition outcomes for women in agriculture: Evidence from rural India
Source: Food Policy. 2021 May;101:102074. doi: 10.1016/j.foodpol.2021.102074 (PMC8214101; doi:10.1016/j.foodpol.2021.102074)
Supplement: Supplementary Data 1 [file mmc1.docx]

**Appendix**

A1: Table showing the composition of household information collected in the household survey

| Men | Household information |
| --- | --- |
|  | household composition |
|  | socioeconomic status |
|  | agricultural practices |
|  | Land use |
|  | livestock |
|  | nonfarm employment |
|  | food availability and access |
|  | empowerment in agriculture |
| Women | agricultural employment |
|  | Nonfarm employment |
|  | semi-quantitative food frequency questionnaire |
|  | home gardens |
|  | household chores |
|  | water |
|  | sanitation |
|  | hygiene |
|  | Health (reproductive history, anemia, and health-seeking behavior) |
|  | child care |
|  | empowerment in agriculture |

A2: Time use variables included in the time use study

| Economically productive work: Field crop farming sector | Travel to farm |
| --- | --- |
|  | Ploughing |
|  | Preparing land |
|  | Cleaning of land |
|  | Sowing |
|  | Planting |
|  | Transplanting |
|  | Application of manure |
|  | Application of fertilizer |
|  | Application of pesticide |
|  | Preparing organic manure |
|  | Harvesting |
|  | Threshing |
|  | Winnowing |
|  | Picking |
|  | Weeding |
|  | Watering |
|  | Supervision of work |
|  | Kitchen garden |
|  | Stocking |
|  | Transportation to home |
|  | Guarding or protection of crop |
|  | Sale and purchase related activities |
|  | Fetching water |
|  | Fetching fuel/twigs/leaves |
|  | Others |
| Economically productive work: Animal husbandry | Grazing animals |
|  | Tending animals – cleaning, preparation of the feed, washing shed, watering |
|  | Caring for animal: breading, grooming, medical |
|  | Poultry rearing – feeding, cleaning |
|  | Sale and purchase related activities |
|  | Fishing |
|  | Forestry Horticulture and Gardening |
|  | Fetching fodder |
|  | Others |
| Economically productive work: Processing and storage work | Milling |
|  | Husking |
|  | Pounding |
|  | Parboiling |
|  | Sorting and grading |
|  | Grinding/Crusting |
|  | Others |
| Economically productive work in secondary sector | Mining |
|  | Food processing and cooking for sale |
|  | Manufacturing of textiles: weaving, knitting etc. |
|  | Making handicrafts |
|  | Construction of own house |
|  | Construction of well/storage facilities/fencing for farm |
|  | Construction of public works: roads, bridges, dams/ MGNAREGA |
|  | Others |
| Economically productive work in tertiary sector | Self employed (Barber, Tailor, Own shop, Contractor, hawking/ vendor, Transport of goods/ people, tuitions) |
|  | Government job |
|  | Private job (driver, guard etc.) |
|  | Others |
| Household management | Cooking food for the HH and serving |
|  | Preparation for cooking |
|  | Cleaning house |
|  | Washing and ironing clothes |
|  | Cleaning utensils |
|  | Shopping for grocery |
|  | Household repairs |
|  | Care for pets |
|  | Fetching of water for HH use |
|  | Fetching of fuel for HH use |
|  | Others |
| Care for children, the sick, elderly and disabled for own HH | Physical care for children: washing, dressing, feeding etc. |
|  | Teaching/ training own children |
|  | Accompanying/travel with children to places (school/PHC/doctor etc.) |
|  | Supervising children with/ without other activity |
|  | Physical care for sick/disabled/elderly: washing, dressing, feeding etc. |
|  | Taking care of guest/ relatives |
|  | Others |
| Community participation and social/cultural activities | Community organized construction and repair: buildings, roads, dams etc. |
|  | Community organized events: cooking for marriages etc. |
|  | Volunteer work |
|  | Participation in meetings : SHG, formal groups, panchayats etc. |
|  | Participation in meetings of informal groups |
|  | Informal help to other HH |
|  | Participating in social events: weddings, funerals etc. |
|  | Participating in religious activities |
|  | Socializing with people at home and outside home |
|  | Others |
| Non-productive work/Personal care and Self- Maintenance | Sleep and related activities |
|  | Eating and Drinking |
|  | Smoking, Drinking, Alcohol, Tobacco and other intoxicants |
|  | Exercise/ Yoga |
|  | Talking/gossiping and quarrelling |
|  | 5 Doing nothing/rest/ relaxation |
|  | Resting due to physical illness |
|  | Personal hygiene and health |
|  | Watching TV/ listening to radio |
|  | Education |
|  | Receiving medical care |
|  | Others |

**A3: Percentage of women working as agricultural labor**

| Did you work as an agricultural labor on someone else's land ? | % |
| --- | --- |
| Yes | 85 |
| No | 15 |

| Variable |  | R1 | R2 |  | R 3 | R 4 | R 5 | R6 | R 7 | R 8 | R 9 | R 10 |
| --- | --- | --- | --- | --- | --- | --- | --- | --- | --- | --- | --- | --- |
| Manufacturing | Mean | 0 | 0 |  | 0 | 6.52 | 0 | 0 | 0 | 0 | 0 | 0 |
|  | N | 21 | 21 |  | 23 | 23 | 23 | 23 | 23 | 23 | 23 | 23 |
| Construction of own house | Mean | 0 | 0 |  | 0 | 0 | 13.04 | 0 | 13.04 | 0 | 20.87 | 0 |
|  | N | 21 | 21 |  | 23 | 23 | 23 | 23 | 23 | 23 | 23.00 | 23 |

**A4: Time spent by women in tertiary and secondary activities across rounds**

| Variable |  | R 1 | R 2 | R 3 | R 4 | R 5 | R 6 | R7 | R 8 | R 9 | R 10 |
| --- | --- | --- | --- | --- | --- | --- | --- | --- | --- | --- | --- |
| Tertiary | Mean | 4.05 | 31.90 | 3.26 | 24.78 | 0.00 | 3.48 | 13.04 | 46.09 | 6.52 | 3.04 |
|  | N | 21 | 21 | 23 | 23 | 23 | 23 | 23 | 23 | 23 | 23 |

*Round 1 = April 2017. Other activities are not represented here since we have 0 values for time use.

**A5: Main results after removing the households that participated in any other income-generating activity besides agriculture (all controls are included).**

|  | (1) | (2) | (3) | (4) | (5) | (6) | (7) |
| --- | --- | --- | --- | --- | --- | --- | --- |
|  | Calories  (Kcal) | Protiens  (g) | Fats  (g) | Iron  (mg) | Zinc  (mg) | Vitamin A  (muG) | mddw10 |
|  | b/se | b/se | b/se | b/se | b/se | b/se | b/se |
| Female wages (Rs./day) | -1.208^***^ | -0.015^***^ | -0.017^***^ | -0.007^***^ | -0.004^**^ | -1.383^***^ | -0.000 |
|  | (0.302) | (0.003) | (0.005) | (0.002) | (0.001) | (0.391) | (0.000) |
| Male wages (Rs./day) | 0.761^**^ | 0.008^**^ | -0.000 | -0.000 | 0.001 | 0.533 | -0.001^***^ |
|  | (0.288) | (0.003) | (0.005) | (0.001) | (0.001) | (0.372) | (0.000) |
| Adj. R-Square | 0.319 | 0.222 | 0.128 | 0.151 | 0.110 | 0.131 | 0.125 |
| N | 7823.000 | 7823.000 | 7823.000 | 7823.000 | 7823.000 | 7823.000 | 7823.000 |

1. All regressions use individual fixed-effect models. Standard errors are clustered at the household level. Significance levels: + 0.10, * 0.05, ** 0.01, *** 0.001.
2. Coefficients represent the specific outcome variable, that is, time spent on the activities. Specification includes male wages, season, and type-of-day dummies, market prices of rice, wheat, onions, potatoes, spinach, tomatoes and pigeon pea, and sickness of adults and children.

**A6: Main results of table 4 (Panel C) after removing standard error clustering**

|  | (1) | (2) | (3) | (4) | (5) | (6) | (7) |
| --- | --- | --- | --- | --- | --- | --- | --- |
|  | Calories  (Kcal) | Protiens  (g) | Fats  (g) | Iron (mg) | Zinc  (mg) | Vitamin A(muG) | mddw10 |
|  | b/se | b/se | b/se | b/se | b/se | b/se | b/se |
| Female wages (Rs./day) | -1.237^***^ | -0.015^***^ | -0.018^***^ | -0.007^***^ | -0.004^***^ | -1.372^***^ | -0.000 |
|  | (0.279) | (0.003) | (0.005) | (0.001) | (0.001) | (0.396) | (0.000) |
| Female wages (Rs./day) | 0.787^**^ | 0.008^**^ | 0.000 | 0.000 | 0.001 | 0.556 | -0.001^***^ |
|  | (0.270) | (0.003) | (0.005) | (0.001) | (0.001) | (0.384) | (0.000) |
| Adj. R-Square | 0.319 | 0.223 | 0.129 | 0.154 | 0.111 | 0.129 | 0.125 |
| N | 8002.000 | 8002.000 | 8002.000 | 8002.000 | 8002.000 | 8002.000 | 8002.000 |
|  |  |  |  |  |  |  |  |
