# Supplementary material for: Seasonal time trade-offs and nutrition outcomes for women in agriculture: Evidence from rural India
Source: Food Policy. 2021 May;101:102074. doi: 10.1016/j.foodpol.2021.102074 (PMC8214101; doi:10.1016/j.foodpol.2021.102074)
Supplement: Supplementary Data 2 [file mmc2.docx]

**Recipe and food code book**

**Tata Cornell Institute for Agriculture and Nutrition**

**Standardized recipes**

| S.# | Food type | Page no. | Code series |
| --- | --- | --- | --- |
| 1 | धान्य (Cereals) | **2** | A0### |
| 2 | कडधान्य (Pulses) | **5** | B0### |
| 3 | पिवळा,छन्द्री भाजीपाला (Orange & red vegetables) | **8** | C0### |
| 4 | मातीत उगवणारे, हिरवा भाजीपाला, कच्चे फळ (केली,पपया) (Roots, green leafy vegetables) | **10** | D/E/G0### |
| 5 | अन्य भाजीपाला (Other vegetables) | **13** | F0### |
| 6 | मटन,चिकन,बदक, अंडे, पाण्यातून मिळणारे पदार्थ' (Non-veg) | **18** | H/I/J0### |
| 7 | दूगदजण्य पदार्थ (Milk products) | **20** | K0### |
| 8 | क्राइड पदार्थ (SNACKS, Home made fried foods) | **22** | N0### |
| 9 | घरी बनविलेले गोड पदार्थ (Home made sweets) | **24** | T0### |
| 10 | Drinks (Hot and Cold) | **27** | Q/R0### |
| 11 | चटणी आणि लोणचे (Chutney’s and pickles) | **29** | V0### |
| 12 | Fruits | **32** | W0### |
| 13 | Vegetables that are eaten raw | **34** |  |
| 14 | Packaged foods | **37** | U0### |

धान्य (Cereals)

| **Code** | **food groups** | **Recipe name** | **Description of recipe** |
| --- | --- | --- | --- |
| A0001 | धान्य | गव्हाची सोजी | सोजी(गहू);पाणी |
| A0002 | धान्य | साधी सोजी | सोजी(गहू);पाणी |
| A0002 | धान्य | फोडणीचा भात | भात;कांदा;टमाटर;तेल |
| A0003 | धान्य | ज्वारीची आंबील | ज्वारीच पीठ;पाणी |
| A0004 | धान्य | तांदळाचा चुरीचा भात | तांदुळ;पाणी |
| A0005 | धान्य | ज्वारीचा कन्या | ज्वारी (कन्या);पाणी |
| A0006 | धान्य | कनकीची पोळी (तेलाची चौपाडी) | कणिक |
| A0007 | धान्य | मिक्स भाकर | ज्वारीच पीठ;उडीद पीठ;पाणी; |
| A0008 | धान्य | तुरीचा डाळीची खिचडी | तांदुळ;डाळ |
| A0009 | धान्य | रोडगा भाकर | गव्हाच पीठ;पाणी;तेल;कांदा |
| A0010 | धान्य | कनकीचा पुऱ्या | कणिक;तेल |
| A0011 | धान्य | गहू,ज्वारी,मिक्स भाकर | कणिक,ज्वारीच पीठ |
| A0012 | धान्य | तांदळाच्या पिठाचीआंबील | तांदळाच पीठ;पाणी |
| A0013 | धान्य | तांदुळ,बेसन मिक्स पापड | तांदळाच पीठ;बेसन;पाणी;तेल |
| A0014 | धान्य | राजमा चावल | राजमा;सिमला मिर्च;कांदा;आलु;फुलकोबी;खडा मसाला;तांदुळ;टमाटर |
| A0015 | धान्य | उकमा,बेसनचकली | उकमा प्याकेटवला,बेसन;तांदळाच पीठ;तेलपाणी |
| A0016 | धान्य | तिखट शेवया | शेवया(कणिक),कांदा;टमाटर;पाणी |
| A0017 | धान्य | धनुल्या | तांदळाच पीठ;पाणी |
| A0018 | धान्य | कणिक,पीठ थालीपीठ | कणिक(गव्हाच पीठ);बेसन;तांदळाच पीठ; |
| A0019 | धान्य | तांदळाच पीठ,बेसन चकली | तांदळाच पीठ;बेसन;तेल;पाणी |
| A0021 | धान्य | आलु;फुलकोबी;वाटाणा भात | आलु;फुलकोबी;टमाटर;वाटाणे(भिजलेले);तांदुळ |
| A0022 | धान्य | व्हेज फुलाव | तांदुळ;फुलकोबी;मिर्ची;सिमला मिर्ची;आलु;टमाटर;मेथी भाजी;गाजर;तेल |
| A0023 | धान्य | अप्पालू वडे | तांदळाच पीठ;चण्याची डाळ;तेल;पाणी |
| A0024 | धान्य | वांग्याचा भाजीचे पराठे | वांग्याची भाजी;गव्हाच पीठ; |
| A0025 | धान्य | कनकीचा तिखट पोळ्या | कणिक |
| A0026 | धान्य | रागीर्याचे शेव | राजगिरा पीठ;पाणी;तेल' |
| A0027 | धान्य | राजगिरा भाकर | राजगिरा पीठ;तेल;पाणी |
| A0028 | धान्य | राजगिरा उकरपेंडी | राजगिर;टमाटर;तेल;पाणी |
| A0029 | धान्य | तांदळाची कनी | तांदुळ; |
| A0030 | धान्य | इडली | तांदुळ;उडीद डाळ |
| A0031 | धान्य | साधा खिचडा | तांदुळ;मुंग डाळ;गहु;लाखोरी डाळ;तुर डाळ;उडीद डाळज्वारीतेल |
| A0032 | धान्य | दोसा | उडदाची डाळ;तांदुळ;दही;पाणी;तेल |
| A0033 | धान्य | खिचडा | ज्वारी;तांदुळ;गहू;कांदा;तुर डाळ;मिर्ची;तेल;मसाला;मसाला पेस्ट पाणी |
| A0034 | धान्य | तांदळाचे आळण | तांदुळ कणिक;मेथी;टमाटर;मिर्ची;तेल |
| A0035 | धान्य | कनकीची उकरपेंडी | कणिक;पाणी |
| A0036 | धान्य | वटाना भात | वटाना;तांदुळ;कांदा;टमाटर;मिर्ची;तेल |
| A0037 | धान्य | कनकीचा रोडगा | कणिक;तेल;पाणी |
| A0038 | धान्य | साधी खिचडी | मुंग दल;तांदुळ;पाणी |
| A0039 | धान्य | तांदळाचा पिठाची उकरपेंडी | तांदळाच पीठ;तेल;पाणी |
| A0040 | धान्य | ज्वारीचा पिठाची उकरपेंडी | ज्वारी पीठ;टमाटर;कांदा;तेल;पाणी |
| A0041 | धान्य | पोळ्याचा चुरमा ( मोकळा ) | कणिक;पोळ्या;कांदा;टमाटर;तेल |
| A0042 | धान्य | मैद्याचा पोळ्या | मैदा;पाणी;तेल |
| A0043 | धान्य | रवा पुरी | रवा;तेल;पाणी |
| A0044 | धान्य | कनकीचा पुऱ्या | कणिक;पाणी;तेल |
| A0045 | धान्य | रव्याचा उपमा | रवा;शेंगदाणे;चना डाळ;कांदा;टमाटर;पाणी;तेल |
| A0046 | धान्य | ढोकळा | तांदुळ;चना डाळ;साखर;तेल |
| A0047 | धान्य | तांदळाचा चकल्या | तांदळाच पीठ;तीळ;तेल |
| A0048 | धान्य | मुंग डाळ खिचडी | तांदुळ;मुंग डाळ;कांदे |
| A0049 | धान्य | शेवल्या ( तिखट ) | शेव्ल्या;कांदा;टमाटर;तेल |
| A0050 | धान्य | पानगे | कणिक;तांदळाच पीठ;पाणी |
| A0051 | धान्य | तांदळाचा पिठाची भाकर | तांदळाच पीठ;पाणी |
| A0052 | धान्य | पुरी | मैदा;पाणी;तेल |
| A0053 | धान्य | भाकरी (बाजरी) | बाजरीच पीठ;तांदळाचपीठ;पाणी |
| A0054 | धान्य | जिरा भात | तांदुळ;पाणी |
| A0055 | धान्य | भाकर | ज्वारीच पीठ; |
| A0056 | धान्य | मसाले भात | तांदुळ;मटर;आलु;मिरची;टमाटर;मीठ;कांदा;तेल;कोबी |
| A0057 | धान्य | पोळी | कणिक |
| A0058 | धान्य | भात | तांदुळ |
| A0059 | धान्य | (वाळलेली) मेथीचे (बोंड) | कणिक |
| A0060 | धान्य | पानगे | कणिक;पाणी |
| A0061 | धान्य | पोपटीचा दाण्याचा भात | पोपटी दाने;टमाटर;कांदा;तेल |
| A0062 | धान्य | दही भात | तांदुळ;दही |
| A0063 | धान्य | उत्तपम | तांदुळ;उडदाची डाळ;टमाटर;तेलपाणी |
| A0064 | धान्य | शिळ्या भाजीचे पराठे | भाजी;कणिक;तांदळाच पीठ;तेल |
| A0065 | धान्य | प्लेन सोजी गव्हाची | सोजी पीठ;पाणी |
| A0066 | धान्य | त्रीपुद्या पोळ्या | कणिक;पाणी |
| A0067 | धान्य | वडी,आलुची भाजी | आलु;मुंगाचा डाळीचा वड्या;टमाटर;ते;पाणी |
| A0068 | धान्य | तुरीचा दाण्याचा भात | तांदुळ;टमाटर;तुरीचे दाने;तेल |
| A0069 | धान्य | रवा,बेसन ढोकळा | रवा;बेसन;दही |
| A0070 | धान्य | ढोकळा (उडीद,चना,तांदुळ) | तांदुळ;चना डाळ;उडीद डाळ |

कडधान्य (Pulses)

| **Code** | **food groups** | **Recipe name** | **Description of recipe** |
| --- | --- | --- | --- |
| B0001 | कडधान्य | नायलोन साबुदाणा | साबुदाणा;तेल;बेसन |
| B0002 | कडधान्य | चना,मुंग,तुर,डाळ फोडणीच वरण | चना;मुंग;तुर;पाणी;मिरची;टमाटर;तेल |
| B0003 | कडधान्य | आलु,चन्याची भाजी | चने;आलु;कांदा;तेल |
| B0004 | कडधान्य | मुंगाचे पकोडे | मुंग डाळ;बेसन; |
| B0005 | कडधान्य | तिळाचा पुऱ्या | तीळ;साखर;शेंगदाणे;खोबर किस;तांदळाच पीठ |
| B0006 | कडधान्य | तुर डाळवडे | तुरडाळ;कांदा;तेल |
| B0007 | कडधान्य | आंबट बेसन | बेसन;दही;कांदा |
| B0008 | कडधान्य | चण्याचा डाळीचे वडे | चना डाळ;कांदा;कणिक |
| B0009 | कडधान्य | बेसन शेव | बेसन;तेल |
| B0010 | कडधान्य | दालिया वडे | दालिया;मैदा;कणिक; |
| B0011 | कडधान्य | मसुर दाळीचे वडे | मसूर डाळ;गव्हाच पीठ;कांदा;तेल |
| B0012 | कडधान्य | शेंगोल्याची भाजी | टमाटर;दही;कणिक;पाणी;तेल |
| B0013 | कडधान्य | चुनवड्याची भाजी | बेसन;पाणी;तेल |
| B0014 | कडधान्य | पोपटीच्या दान्याच आळण | पोपटीचे दाने;टमाटर;कांदा;मिर्ची;पाणी;तांदळाच पीठ |
| B0015 | कडधान्य | साबुदाणा वडा | साबुदाणा;मिर्ची;शेंगदाणे;कांदा;तीळ;सांबार;कणिक;तेल |
| B0016 | कडधान्य | बेसन,रवा बर्फी | बेसन;डालडा;साखर;पाणी |
| B0017 | कडधान्य | तांदुळ,वाटणा,फुलकोबी,सोयाबीन वड्या,भात | तांदुळ;वाटणा;फुलकोबी;सोयाबीन वडी;टमाटर;तेल;पाणी |
| B0018 | कडधान्य | लाखोरीचा डाळीचे तिखट वरण | लाखोरीची डाळ;तेल;पाणी |
| B0019 | कडधान्य | उडदाचा डाळीचे सुकवडे (तिखट) | उडदाची डाळ;कणिक;पाणी;तेल |
| B0020 | कडधान्य | उडदाची डाळीचे भजे | उडदाची डाळ;गव्हाच पीठ;पालक;सांबार;तेल |
| B0021 | कडधान्य | शेव भाजी | शेव;टमाटर;कांदा;पाणी;तेल |
| B0022 | कडधान्य | वाळलेल्या वाटण्याची रस्याची भाजी | वाटणा;कांदा;टमाटर;पाणी;तेल |
| B0023 | कडधान्य | वाटण्याचे उसळ | वाटणा;कांदा |
| B0024 | कडधान्य | तुरीचे दान्याच उसळ | तुरी;कांदा;टमाटर |
| B0025 | कडधान्य | बरबटी दाण्याचे वडे | बरबटी;तेल |
| B0026 | कडधान्य | तुर दाण्याचे आळण | तुर दाने;तेल;मिर्ची;टमाटर;पाणी |
| B0027 | कडधान्य | तुरीचा घुगऱ्या(साध्या) | तुरी;कांदा;टमाटर |
| B0028 | कडधान्य | बरबटीचे उसळ | बरबटी;टमाटर;तेल |
| B0029 | कडधान्य | सांबार(इडली करिता) | तुर डाळ;मुंगडाळ;पाणी |
| B0030 | कडधान्य | कबुली चना व आलुची भाजी | आलु;कबुली चना;कांदा;टमाटर |
| B0031 | कडधान्य | तुर डाळ तिखट वरण | तुर डाळ;टमाटर, |
| B0032 | कडधान्य | मुंग डाळ रस्याची भाजी | मुंग डाळ;कांदा;तेल |
| B0033 | कडधान्य | उडदाचा डाळीचे सुकोडे | उडदाची डाळ;कणिक;पाणी;तेल |
| B0034 | कडधान्य | मुंग डाळीचे तिखट वरण | मुंग डाळ;टमाटर;पाणी |
| B0035 | कडधान्य | बरबटी,आलुची भाजी | आलु;बरबटी;टमाटर;तेल |
| B0036 | कडधान्य | लाखोरीचा हिरव्या दाण्याचे आळण | लाखोरीचे हिरवे दाने;मिर्ची;टमाटर;तेल;तांदळाचपीठ;पाणी' |
| B0037 | कडधान्य | तुर डाळ(वरण) साध | तुर डाळ;पाणी |
| B0038 | कडधान्य | खाकस,खोबर,शेंगदाणे,दारल्या भाजी | खोबर;शेंगदाणे;दारल्या;टमाटर;मिरची;तेल |
| B0039 | कडधान्य | डाळ कांदा | चना डाळ;कांदा;ओला कांदा;टमाटर;तेल |
| B0040 | कडधान्य | छोला मसाला | छोले (चना);तेल;खडा मसाला |
| B0041 | कडधान्य | तुरीचे उसळ | तुरी;मिरची;कांदा;मीठ;टमाटर;तेल;पाणी |
| B0042 | कडधान्य | मुंगाचे वडे | मुंग;कांदा;कणिक |
| B0043 | कडधान्य | बारबटी | बरबटी;कांदा;टमाटर;तेल;पाणी |
| B0044 | कडधान्य | मोटच उसळ | मोट;कांदा;टमाटर |
| B0045 | कडधान्य | पाटवडीची भाजी | बेसन;पालक पेस्ट;(टमाटर ;कांदा ;खडा मसाला पेस्ट);तेल;अद्रक लसून पेस्ट |
| B0046 | कडधान्य | मोटच उसळ | मोट;ओली मिरची;टमाटर;कांदे;तेल |
| B0047 | कडधान्य | मुंगदाळीची वडे | मुंग डाळ;कांदे;बेसन |
| B0048 | कडधान्य | भजे | बेसन; |
| B0049 | कडधान्य | फोडणीचे वरण | तुरीची डाळ;मिर्ची |
| B0050 | कडधान्य | लाखाचा डाळीचे वडे | लाखाची डाळ |
| B0051 | कडधान्य | मोकळे बेसन | बेसन;टमाटर;कांदा;मिरची;तेल;मीठ;ओला कांदा;पाणी |
| B0052 | कडधान्य | सोयाबीन वडी भाजी | सोयाबीन वडी;टमाटर;तेल; |
| B0052 | कडधान्य | भज्याची भाजी | भजे; |
| B0053 | कडधान्य | बेसन,कणिक भाकर | बेसन;कणिक;कांदा;टमाटर;तेल;पाणी |
| B0054 | कडधान्य | कच्चा बेसनाचे पराठे | बेसन;पाणी;तेल |
| B0055 | कडधान्य | मोटचा डाळीचे वडे | मोटची डाळ;मिरची;कांदे;तेल;तांदळाच पीठ;सांभार;कणिक;पाणी |
| B0056 | कडधान्य | फोडणीचा वरणातले शेंगुळे | तुर डाळ;ज्वारीच पीठ;टमाटर;कांदा |
| B0057 | कडधान्य | दही वडे | उडदाची डाळ;दही;तेल;पाणी |
| B0058 | कडधान्य | अप्पे | तांदुळ;मुंग डाळ;तुर डाळ;उडीद डाळ;चना डाळ; |

पिवळा, छन्द्री भाजीपाला (Orange and red vegetables)

| C0001 | पिवळा,छन्द्री भाजीपाला | काकडी,गाजर,मुळा चटणी | गाजर;मुळा;काकडी;टमाटर;कांदा,मिर्ची,तेल |
| --- | --- | --- | --- |
| C0002 | पिवळा,छन्द्री भाजीपाला | मेथी;पपई;सांभार वडी | पपई;तांदळाच पीठ;तेल |
| C0003 | पिवळा,छन्द्री भाजीपाला | पपईचे बोंड | पपई;कणिक;साखर;तेल |
| C0004 | पिवळा,छन्द्री भाजीपाला | पपईचे वडे | तांदळाच पीठ;तेल |
| C0005 | पिवळा,छन्द्री भाजीपाला | सलाद | मुळा;गाजर |
| C0006 | पिवळा,छन्द्री भाजीपाला | मुळ्याचे वडे | मुळे;मिर्ची;तांदळाच पीठ;तेल |
| C0007 | पिवळा,छन्द्री भाजीपाला | चण्याची डाळ,कोहळभाजी | कोहळ;चना डाळ;टमाटर |
| C0008 | पिवळा,छन्द्री भाजीपाला | कोहळ,वाटाना भाजी | कोहळ;टमाटर;मिर्ची;वाटाणा;पाणी;तेल |
| C0009 | पिवळा व छन्द्री भाजीपाला | कोवळ्याची भाजी | कोवळ;टमाटर;कांदा;सांबार;तेल |
| C0010 | पिवळा,छन्द्री भाजीपाला | काकडीची भाजी | काकडी;टमाटर;तेल |
| C0011 | पिवळा,छन्द्री भाजीपाला | बीटचे पराठे | बीट;मेथी;बेसन;कणिक;पालक;तांदळाच पीठ;मिरची पेस्ट;पाणी |
| C0012 | पिवळा,छन्द्री भाजीपाला | रात्नाळचे पुरण | रतनाळ;साखर;पाणी;तेल |
| C0013 | पिवळा,छन्द्री भाजीपाला | काकडीचे भजे | काकडी;बेसन;कांदा;तेल |
| C0014 | पिवळा,छन्द्री भाजीपाला | कोव्ल्याचे बोंड | कोवळ;पाणी;तांदळाच पीठ;तेल;कणिक |
| C0015 | पिवळा,छन्द्री भाजीपाला | दुधीचे वडे | दुधी;तेल;तांदळाचपीठ; |
| C0016 | पिवळा,छन्द्री भाजीपाला | काकडीचे पराठे | काकडी;कणिक;तेल |
| C0017 | पिवळा,छन्द्री भाजीपाला | फणसची भाजी | फणस;टमाटर;तेल |
| C0018 | पिवळा,छन्द्री भाजीपाला | मुळ्याचे वडे | मुळा;पाणी |

मातीत उगवणारे, हिरवा भाजीपाला, कच्चे फळ (केली,पपया) (Roots, green leafy vegetables)

| D0001 | मातीत उगवणारे | कच्चा आलूची भाजी | आलू;तेल;टमाटर;पाणी |
| --- | --- | --- | --- |
| D0002 | मातीत उगवणारे | समोसे | आलु;वाटाना;टमाटर;मैदा;तेल;पाणी |
| D0003 | मातीत उगवणारे | आलु,वाटाना भाजी | आलु,वाटाणा,तेल;टमाटर;कांदा;पाणी |
| D0004 | मातीत उगवणारे | आलु कोप्ता | आलु;कांदा |
| D0005 | मातीत उगवणारे | करी ( कोपता ) | आलु |
| D0006 | मातीत उगवणारे | आलु रस्याची भाजी | आलु;टमाटर;तेल |
| D0007 | मातीत उगवणारे | आलु पराठा | आलु;कणिक;तेल |
| D0008 | मातीत उगवणारे | भाजलेल्या आलुची भाजी | आलु;कांदा;तेल;पाणी |
| E0001 | हिरवा भाजीपाला | पालक भजे | पालक;बेसन;कांदे;तेल |
| E0002 | हिरवा भाजीपाला | सांबार वडी | सांबार;खोबर;तांदळाच पीठ;तीळ |
| E0003 | हिरवा भाजीपाला | मेथी,आलु भाजी | आलु;कांदा;टमाटर;मेथी;पाणी |
| E0004 | हिरवा भाजीपाला | पालक भाजी मोकळी | पालक;टमाटर |
| E0004 | हिरवा भाजीपाला | धोप्याचा पानाची डाळभाजी | तुर;डाळ;चना डाळ;टमाटर;धोप्याची पाने;कांदा |
| E0005 | हिरवा भाजीपाला | बेसन,पालक पराठे | बेसन;पालक;कणिक; |
| E0006 | हिरवा भाजीपाला | पालक पुरी | पालक;कणिक;तेल;पाणी |
| E0007 | हिरवा भाजीपाला | राजगिर्याची मोकळी भाजी | राजगिर्याची भाजी |
| E0008 | हिरवा भाजीपाला | बटव्याची भाजी | बटव्याची भाजी;कांदा;मिर्ची |
| E0009 | हिरवा भाजीपाला' | सांबार वडी | बेसन,सांबर,कांदा,तेल;हिरवी वाटाणे |
| E0010 | हिरवा भाजीपाला | पालक,आलूची भाजी | पालक;आलु;टमाटर;तेल |
| E0011 | हिरवा भाजीपाला | मेथी पुरी | मेथी भाजी;कणिक;कांदा;कांदा,सांबार पेस्ट;तेल |
| E0012 | हिरवा भाजीपाला | कुडकुडीचे मुत्ठे | कुडकुडीची भाजी;तांदळाच पीठ;कांदा;तेल;टमाटर;पाणी |
| E0013 | हिरवा भाजीपाला | कांदा ओल्या पालीचे वडे | कांदा चोप;तांदळाच पीठ;पाणी |
| E0014 | हिरवा भाजीपला | ओल्या कांद्याचे आयते | ओलाकांद्याचा पाला;मिर्ची;तांदळाचपीठ;पाणी;तेल |
| E0015 | हिरवा भाजीपाला | पालकचे आयते | तांदुळ पीठ;पालक;गव्हाच पीठ;मिर्ची;कांदा;तेल |
| E0016 | हिरवा भाजीपाला | घोळणा कच्चा | हिरवी मेथी;टमाटर;कांदा;हिरवी मिर्ची |
| E0017 | हिरवा भाजीपाला | कुद्कुडीचे आयते | कुद्कुडीची भाजी;तांदळाच पीठ;पाणी';तेल |
| E0018 | हिरवा भाजीपाला | पालक,मेथी पुरी | पालक;मेथी;गव्हाच पीठ;पाणी;तेल |
| E0019 | हिरवा भाजीपाला | मेथी,आलु पराठा | आलु;मेथी;सांबार;ओला कांदा;बेसन;कणिक |
| E0020 | हिरवा भाजीपाला | पालक-तुरीची डाळभाजी | पालक;तुरीची डाळ;टमाटर |
| E0021 | हिरवा भाजीपाला | घोळची भाजी | घोळ;टमाटर;कांदा;तेल |
| E0022 | हिरवा भाजीपाला | मेथीचे आयते | मेथी;तांदळाच पीठ;मिर्ची;पाणी;साखर;तेल |
| E0023 | हिरवा भाजीपाला | पालक,आलुची भाजी' | पालक;आलु;कांदा;टमाटर;तिखट;मीठ;तेल;पेस्ट पाणी |
| E0024 | हिरवा भाजीपाला | हरभर्याची हिरवी भाजी | हरभरा भाजी;टमाटर;तेल |
| E0025 | हिरवा भाजीपाला | ओल्या मेथीची भाजी | ओलीमेथी;कांदा;मिरची;टमाटर;तुरीचे दाने;तेल |
| E0026 | हिरवा भाजीपाला | पालक पराठा | पालक;कणिक;बेसन;टमाटर;कांदा;तेल |
| E0027 | हिरवा भाजिपाला | मेथीचे पराठे | मेथी;सांभार;पाणी;कणिक |
| E0028 | हिरवा भाजीपाला | पालक,डाळभाजी | पालक;तुर डाळ;चना डाळ;टमाटर;तेल |
| E0029 | हिरवा भाजीपाला | आयते लसणाचे | लसणाचा पाला;मिरची;तांदळाच पीठ;पालक;मेथी;तेल;पाणी |
| E0030 | हिरवा भाजीपाला | सुरुंग कांद्याची भाजी | तेल;मसाला;सुरुंग;पाणी;कांदा;टमाटर |
| E0031 | हिरवा भाजीपाला | आलु,चना भाजी हिरवी पाने | आलु;चना भाजी;टमाटर;तेल;पाणी |
| E0032 | हिरवा भाजीपाला | शेपूची भाजी | शेपू;मुंगाची डाळ; |
| E0033 | हिरवा भाजीपाला | राइची मोकळी भाजी | रे हिरवी;टमाटर |
| E0034 | हिरवा भाजीपाला | आंबट चुका डाळभाजी,मुंग डाळ | आंबटचुका भाजी;मुंग डाळ;टमाटर |
| E0035 | हिरवा भाजीपाला | आंबट चुका डाळभाजी | आंबट चुका;तुरीची डाळ;मुंगाची डाळ;चना डाळ |
| E0036 | हिरवा भाजीपाला | चवळीची भाजी मोकळी | चवळीची भाजी |
| E0037 | हिरवा भाजीपाला | चवलीची भाजी,आलु | चवळीची भाजी;आलु;टमाटर |
| E0038 | हिरवा भाजीपाला | वाळली मेथी भाजी (सुखी भाजी) | मेथी (बीज);बाजारातले;टमाटर |
| E0039 | हिरवा भाजीपाला | धोप्याचा पानाची डाळभाजी | मुंड डाळ;तुर डाळ;मसुर डाळ;धोप्याचे पान;तेल;पाणी |
| E0040 | हिरवा भाजीपाला | धोप्याचा पानाचे वडे | धोपा;बेसन;तेल;पाणी |
| E0041 | हिरवा भाजीपाला | धोप्याची वडी भाजी | धोपा पान;बेसन;कांदा;टमाटर |
| E0042 | हिरवा भाजीपाला | धोप्याची वडी | धोपा पान;बेसन |
| E0043 | हिरवा भाजीपाला | मोकळे बेसन धोपा | धोपा;कांदा;बेसन |
| G0001 | कच्चे फळ (केली,पपया) | पपईची भाजी | पपई;टमाटर;कांदा;तेल |
| G0002 | कच्चे फळ (केळी,पपया) | कच्चा केळाची रस्स्याची भाजी | कच्ची केळी;तेल;सांबार;पाणी |
| G0003 | कच्चे फळ (केळी,पपया) | केळीची भजी कच्ची | कच्ची केळी;बेसन;पाणी;तेल |
| G0004 | कच्चे फळे(केळी,पपया) | कच्चा केळाची भाजी | कच्ची केळी;टमाटर;पाणी |

अन्य भाजीपाला (Other vegetables)

| **Code** | **food groups** | **Recipe name** | **Description of recipe** |
| --- | --- | --- | --- |
| F0001 | अन्य भाजीपाला | आलू बोंडे | आलु;पाणी;बेसन;मिरची;टमाटर;तेल;कांदे |
| F0002 | अन्य भाजीपाला | मसाला तोंडरे | तोंडरे;टमाटर; |
| F0003 | अन्य भाजीपाला | चवळीचा शेंगा (काळ्या) भाजी | चवळीचा शेंगा,टमाटर, |
| F0004 | अन्य भाजीपाला | सिमला मिर्ची,आलुची भाजी | सिमला मिर्ची;आलु;मिर्ची;टमाटर;कांदा;तेल;पाणी |
| F0005 | अन्य भाजीपाला | पत्ताकोबीचे भजे | पत्ताकोबी;मिर्ची;कांदा;बेसन(चना डाळ);तेल;पाणी |
| F0006 | अन्य भाजीपाला | डाळ वांग्याची भाजी | वांगे;मिर्ची;टमाटर;मुंग डाळ;तुर डाळ;;मीठ;तेल;मेथी |
| F0007 | अन्य भाजीपाला | पत्ताकोबीचे पराठे | पत्ताकोबी;मिर्ची;मेथी;तांदळाच पीठ;पाणी;तेल |
| F0008 | अन्य भाजीपाला | फुलकोबी,चन्याची भाजी | फुलकोबी;हिरवे चने;तेल;कांदा;टमाटर |
| F0009 | अन्य भाजीपाला | आलुचे भजे | आलु,बेसन,तेल |
| F0010 | अन्य भाजीपाला | दम आलु | आलु;टमाटर; |
| F0011 | अन्य भाजीपाला | फुलकोबीचा पाल्याची भाजी | फुलकोबी पाने;टमाटर;तेल;पाणी |
| F0012 | अन्य भाजीपाला | पत्ताकोबीचा आलुची भाजी | पत्ताकोबी;आलु;टमाटर;कांदा;तेल;पाणी;संभार;मेथी |
| F0013 | अन्य भाजीपाला | वटण्याचा शेंगाची भाजी | वाटनाच्या शेंगाच;कांदा;टमाटर |
| F0014 | अन्य भाजीपाला | पत्ताकोबीचे वडे | पत्ताकोबी;मिर्ची;कांदा;तांदळाच पीठ;तेल;पाणी |
| F0015 | अन्य भाजीपाला | लवकीची भाजी | लवकी;कांदा;तेल |
| F0016 | अन्य भाजीपाला | आलुची चटणी | आलु;टमाटर;कांदा;तेल |
| F0017 | अन्य भाजीपाला | लवकीचे पराठे | लवकी;सांबार;मिर्ची;कांदा;मेथी;तांदळाच पीठतेल |
| F0018 | अन्य भाजीपाला | काकडीचे पराठे लाटुन | काकडी;कणिक |
| F0019 | अन्य भाजीपाला | चना डाळ,पत्ताकोबीची भाजी | पत्ताकोबी;चना डाळ;कांदा;टमाटर |
| F0020 | अन्य भाजीपाला | मिर्ची फोलाची भाजी | मिरची;टमाटर |
| F0021 | अन्य भाजीपाला | कांदा भजे | कांदा;बेसन;तेल;पाणी |
| F0022 | अन्य भाजीपाला | वांगे, वालाचा शेंगाची भाजी | वालाचा शेंगा;वांगे;टमाटर |
| F0023 | अन्य भाजीपाला | भेंडी भजे | भेंडी;बेसन;तांदळाच पीठ;तेल |
| F0024 | अन्य भाजीपाला | पत्ताकोबी ,आलुची,हिरवा वटानाची भाजी | पत्ताकोबी;आलु;हिरवा वाटणा;पाणी |
| F0025 | अन्य भाजीपाला | वालाचा शेंगाचे मुत्ठे (कुडमुड) | वालाचा शेंगा;टमाटर;तांदळाच पीठ;तेल;पाणी |
| F0026 | अन्य भाजीपाला | वांगे, वालाचा शेंगाचा दाण्याची भाजी | वांगे;वालाचा शेंगा;टमाटर |
| F0027 | अन्य भाजीपाला | वांग्याचा खुलाची भाजी | वांग्याचा खुला;कांदा;तेल;टमाटर |
| F0028 | अन्य भाजीपाला | भेंडीच्या दान्याच आळण | भेंडीचे दाने';कांदा;टमाटर;तांदळाचपीठ;तेल;पाणी |
| F0029 | अन्य भाजीपाला | कोबी,आलु भाजी | फुलकोबी,आलु;कांदा;टमाटर |
| F0030 | अन्य भाजीपाला | भेंडीचा दाण्याचे वडे | भेंडीचे दाने;तांदळाच पीठ;पाणी;तेल |
| F0031 | अन्य भाजीपाला | हिरव्या मिरची,वांग्याची भाजी | वांगे;तेल;कांदा;टमाटर |
| F0032 | अन्य भाजीपाला | वालाचा शेंगाचा दाण्याचे आळण | वालाचा शेंगाचे दाने;टमाटर |
| F0033 | अन्य भाजीपाला | तुरीचे दाने,वांग्याची भाजी | वांगे;तुरीचे दाने;टमाटर;कांदा;तेल |
| F0034 | अन्य भाजीपाला | तुरीचा दाण्याचे वडे | तुरीचे दाने;हिरवी मिर्ची;तांदळाच पीठ;तेल |
| F0035 | अन्य भाजीपाला | हिरव्या चण्याचे वडे | चना (हिरवा );कणिक |
| F0036 | अन्य भाजीपाला | हिरव्या चण्याची भाजी | हिरवा चना;टमाटर; |
| F0037 | अन्य भाजीपाला | दही मिर्ची | मिर्ची;दही |
| F0038 | अन्य भाजीपाला | तुरीचा दाण्याचे आळण | तुरीचे दाने;टमाटर;कांदा;मिर्ची;तेल;अद्रक-लसून-जीर पेस्ट,अद्रक |
| F0039 | अन्य भाजीपाला | मसाला भेंडी | भेंडी;ओला मसाला;पेस्ट पाणी;तेल |
| F0040 | अन्य भाजीपाला | टमाटरचे भजे | टमाटर;बेसन;तेल;पाणी |
| F0041 | अन्य भाजीपाला | वांगे आणि चवळीचा शेंगा | चवळीचा शेंगा;टमाटर;वांगे;तेल;पाणी |
| F0042 | अन्य भाजीपाला | पत्तकोबी,मुंग डाळ | पत्तकोबी;कांदा;टमाटर;मुंग डाळ;तेल |
| F0043 | अन्य भाजीपाला | सिमला मिर्च,बेसन | सिमला मिर्च;कंद;टमाटर;तेल;बेसन;पाणी |
| F0044 | अन्य भाजीपाला | वांगे,मेथी भाजी | वांगे;मेथी;टमाटर |
| F0045 | अन्य भाजीपाला | वालाचा शेंगाचा खुलाची भाजी' | वालाचा खुला;टमाटर; |
| F0046 | अन्य भाजीपाला | पोपटीचे हिरवे दाने,तुरीचे हिरवे दान्याच आळण | पोपटीचे दाने;टमाटर;तुरीचे दाने |
| F0047 | अन्य भाजीपाला | पोपट दाने,वांगे भाजी | पोपट दाने;वांगे;मिर्ची;कांदा;टमाटर;तेल |
| F0048 | अन्य भाजीपाला | तोंडरे तुरडाळ भाजी | तोंडरे;तुरीची डाळ;टमाटर;सांभार;तेल;पाणी |
| F0049 | अन्य भाजीपाला | चवळीचा,आलूचा शेंगाची भाजी | आलु;चवळीचा शेंगा;टमाटर;कांदा;सांभार;तेल |
| F0050 | अन्य भाजीपाला | फुलकोबीचे भजे | फुलकोबी;मेथी;पलेचा कांदा;मिरची;हळद;तेलबेसन;पाणी |
| F0051 | अन्य भाजीपाला | वांगे,फुलकोबी भाजी | वांगे;फुलकोबी;टमाटर;तेल |
| F0052 | अन्य भाजीपाला | फणसाचा बियांची भाजी | फणस बिया;कांदा;टमाटर;तेल |
| F0053 | अन्य भाजीपाला | मसाल्याचे कारले | कारले ;मिर्ची;कांदा;टमाटर;तेल |
| F0054 | अन्य भाजीपाला | तोंडरीची भाजी,पोपटीचे दाने | तोंडरे;पोपटीचे दाने;टमाटर;कांदा;तेल |
| F0055 | अन्य भाजीपाला | शिमला मिर्ची | शिमला मिर्ची;कांदा;टमाटर |
| F0056 | अन्य भाजीपाला | पोपटीचा दाण्याची भाजी | पोपटीचे दाने;कांदा;टमाटर;तेल |
| F0057 | अन्य भाजीपाला | कांद्याचे चोप्चे भजे | कांद्याची चोप;मिरची;तेल;बेसन |
| F0058 | अन्य भाजीपाला | मसाला वांगे | वांगे;टमाटर;कांदे;मसाला पेस्ट;पाणी;तेल |
| F0059 | अन्य भाजीपाला | भेंडीची भाजी | भेंडी;कांदा |
| F0060 | अन्य भाजीपाला | चवळीचा शेंगाची भाजी | चवळीचा शेंगा;कांदा;टमाटर;तेल |
| F0061 | अन्य भाजीपाला | पत्ताकोबीची भाजी | पत्तकोबी |
| F0062 | अन्य भाजीपाला | फुलकोबीची भाजी | फुलकोबी;कांदा;टमाटर |
| F0063 | अन्य भाजीपाला | गवार शेंगा भाजी | गवार शेंगा;शेंगदाणे;टमाटर;कांदा |
| F0064 | अन्य भाजीपाला | वालाचा शेंगा | वालाचा शेंगा;कांदा;टमाटर; |
| F0065 | अन्य भाजीपाला | कारल्याची भाजी | कारले ;मिर्ची;कांदा;टमाटर |
| F0066 | अन्य भाजीपाला | वांग्याचं भरीत | वांगे;टमाटर; ओला कांदे; |
| F0067 | अन्य भाजीपाला | आलु,कोबी,मटरची भाजी | आलु;फुलकोबी;वाटाणे;टमाटर;कांदा;मिरची;तेल |
| F0068 | अन्य भाजीपाला | आलु वांग्याची भाजी | वांगे;आलु;टमाटर;लसून,कांदा पेस्ट |
| F0069 | अन्य भाजीपाला | टमाटर कढी | टमाटर;मीठ;तेल;पाणी |
| F0070 | अन्य भाजीपाला | दोडक्याची भाजी | दोडके |
| F0071 | अन्य भाजीपाला | तुरीचे दाने (करंजी ) रस्सा | तुरीचे दाने;कणिक;तेल |
| F0072 | अन्य भाजीपाला | पोपटीचा दाण्याची सुखी भाजी | पोपटीचे दाने;टमाटर;तेल |
| F0073 | अन्य भाजीपाला | चवळीचे दाने मिक्स भाजी | चवळी दाने;आलू;वांगे;टमाटर;कांदा;पाणी |
| F0074 | अन्य भाजीपाला | पर्वेल्ची भाजी | परवेल;कांदा |
| F0075 | अन्य भाजीपाला | मसाले दाने भाजी (चवळी) | चवळीचे दाने;टमाटर;कांदा;तेल |
| F0076 | अन्य भाजीपाला | दोडके भाजी (रस्सा) | दोडके;टमाटर |
| F0077 | अन्य भाजीपाला | केळी वांगे,अअळूची भाजी | वांगे;आलु;कांदा;टमाटर;लासुन पेस्ट;तेल;पाणी |
| F0078 | अन्य भाजीपाला | चाव्लीचा दाण्याचे आळण | चवळीचे दाने;टमाटर;कांदा;तेल |
| F0079 | अन्य भाजीपाला | परसबिन शेंगा | परसबिन शेंगा;टमाटर |
| F0080 | अन्य भाजीपाला | चवळीचा दाण्याचे वडे' | चवळीचे दाने'तांदळाच पीठ |
| F0081 | अन्य भाजीपाला | दोडका डाळ भाजी (चना,तुर डाळ) | दोडके;चना तुर डाळ;तुर डाळ;टमाटर;पाणी |
| F0082 | अन्य भाजीपाला | मसाला गवार शेंगा | गव्हार शेंगा;तेल;टमाटर |
| F0083 | अन्य भाजीपाला | चवळीचे दाने भाजी (मोकळी) | चवळी दाने;टमाटर |
| F0084 | अन्य भाजीपाला | भेंडीची साधी भाजी | भेंडी;तेल |
| F0085 | अन्य भाजीपाला | दोडके भजे | दोडके;बेसन;कांदा;तेल;पाणी |
| F0086 | अन्य भाजीपाला | लाल भाजी(माट)(चवळी) | लाल भाजी;कांदे |
| F0087 | अन्य भाजीपाला | गवार,तीळ भाजी | गवार शेंगा;टमाटर |
| F0088 | अन्य भाजीपाला | लांब दोडका,मुंग डाळ भाजी | दोडका,मुंग डाळ;टमाटर;तेल |
| F0089 | अन्य भाजीपाला | गवार,आलुची भाजी | गवार;आलु;टमाटर |
| F0090 | अन्य भाजीपाला | मुंग्ण्याचा शेंगाची भाजी | मुंग्न्याचा शेंगा;कांदा;टमाटर;तेल;खडा मसाला;पानि |
| F0091 | अन्य भाजीपाला | नवरगोल | नवरगोल;तेल |
| F0092 | अन्य भाजीपाला | मीक्स भाजी | ओळ वटणा;शिमला मिर्ची;वालाचा शेंगा;फुलकोबी;वांगे;आलु;टमाटर;सांबार,कांदा |
| F0093 | अन्य भाजीपाला | भगर | भगर;आलु;टमाटर; |
| F0094 | अन्य भाजीपाला | चिंचेची कडी | चिंच;गुळ;साखर;मीठ;पाणी;कांदे |
| F0095 | अन्य भाजीपाला | आमचूर कडी | आमचूर |
| F0096 | अन्य भाजीपाला | वाळलेल्या मेथीची भाजी | मेथी;कांदा;टमाटर;सांभार |
| F0097 | अन्य भाजीपाला | पालक,पनीर,सोयाबीन वाडी भाजी | सोयाबीन;पालक;पनीर;कांदा;टमाटर;सांभार |

मटन,चिकन,बदक, अंडे, पाण्यातून मिळणारे पदार्थ' (Non-veg)

| **Code** | **food groups** | **Recipe name** | **Description of recipe** |
| --- | --- | --- | --- |
| H0001 | मटन,चिकन,बदक | झिंगा भाजी | झिंगे;खडा मसाला;टमाटर;पाणी |
| H0002 | मटन,चिकन,बदक,इ. | चिकन | चिकन;लसून,अद्रक पेस्ट;मसाला पेस्ट(खोबर,भेंडी विलायची,मिरी,कलमी,खाकस) |
| H0003 | मटन,चिकन,बदक,इ. | मटन बकऱ्याच | मटन;कांदा;खडा मसाला;तेल;पाणी |
| H0004 | मटन,चिकन,बदक,इ. | चिकन बिर्याणी | तांदुळ;चिकन;तेल;पाणी |
| H0005 | मटन,चिकन,बदक,इ. | गावरानि चिकन | चिकन;लसून;तेल;लसून,जीर,अद्रक पेस्ट;पाणी |
| I0001 | अंडे | अंडा भजे | अंडे;बेसन;पाणी;तेल; |
| I0002 | अंडे | अंड्याचे धापुडे | अंडे;कांदा |
| I0003 | अंडे | अंडा पनीर भाजी | कांदा;टमाटर;अंडी;पेस्ट मसाला पाणी;पाणी; |
| I0004 | अंडे | अंडा भात | तांदुळ;पाणी;अंड्याचा गार;कांदा;टमाटर;तेल |
| I0005 | अंडे | ब्रेड ऑमलेट | अंडे;कांदे;तेल;ब्रेड |
| I0006 | अंडे | ऑमलेट | कांदे;अंडे;तेल |
| I0007 | अंडे | बॉईल अंडे | अंडे;पाणी |
| I0008 | अंडे | अंडा बिर्याणी | अंडे;तांदुळ;पाणी;तेल; |
| I0009 | अंडे | मेथी अंडा भुर्जी | मेथी;अंड्याचा गर;हिरवी मिर्ची';टमाटर;कांदा;तेल |
| I0010 | अंडे | अंडा भुर्जी | अंडे;कांदे;टमाटर;तेल |
| I0011 | अंडे | अंडा भाजी | अंडे;कांदा;मसाला पेस्ट(लसून,अद्रक,खाकस,जीर,शेंगदाणा);पेस्ट पाणी |
| I0012 | अंडे | अंडा ब्रेंड पकोडा | अंडे;बेसन;ब्रेड |
| J0001 | पाण्यातून मिळणारे पदार्थ' | मछीची भाजी ( FISH CURRY ) | मासोळी;लसून;खडा मसाला;कांदा;टमाटर;तेल |

दूगदजण्य पदार्थ (Milk products)

| **Code** | **food groups** | **Recipe name** | **Description of recipe** |
| --- | --- | --- | --- |
| K0001 | दूगदजण्य पदार्थ | श्रीखंड | दही;साखर |
| K0002 | दुग्दजन्य पदार्थ | दही पुरी | गव्हाचे पीठ;रवा;साखर;दही;तेल;पाणी |
| K0003 | दुग्धजन्य पदार्थ | मसाला दुध | दुध;साखर |
| K0004 | दुग्धजन्य पदार्थ | पनीरची भाजी | पनीर;तेल;पाणी |
| K0005 | दुग्धजन्य पदार्थ | बासुंदी | दुध;साखर |
| K0006 | दुग्धजन्य पदार्थ | कडी ( ताकाची ) | ताक |
| K0007 | दुग्धजन्य पदार्थ | कडी | दही |
| K0008 | दुग्धजन्य पदार्थ | रबडी | दुध;साखर |
| K0009 | दुग्धजन्य पदार्थ | शाही पनीर | पनीर;तूप;कांदा;टमाटर;तेल |
| K0010 | दुग्धजन्य पदार्थ | दही कलाकंद | दही;साखर |
| K0011 | दुग्धजन्य पदार्थ | मटठा | दही;पाणी |
| K0012 | दुग्धजन्य पदार्थ | पालक पनीरची भाजी | पनीर;पालक;टमाटर;तेल;पाणी |
| K0013 | दुग्धजन्य पदार्थ | कलाकंद | दुध;साखर |
| K0014 | दुग्धजन्य पदार्थ | दुधाचा पेडा | दुध;साखर |
| K0015 | दुग्धजन्य पदार्थ | दही लस्सी | दही;साखर;पाणी |

क्राइड पदार्थ (Fried foods: Snacks)

| **Code** | **food groups** | **Recipe name** | **Description of recipe** |
| --- | --- | --- | --- |
| N0001 | (SNACKS) क्राइड पदार्थ | खारे शंकरपाळे | मैदा;पाणी;तेल |
| N0002 | (SNACKS) क्राइड पदार्थ | शेंगदाणे वडे | शेंगदाणे पाणी |
| N0003 | (SNACKS) क्राइड पदार्थ | भेल | मुरमुरे;पातळ पोहे;शेंगदाणे;दलिया;कांदा;मिर्ची;शेव चिवडा;तेल;टमाटर |
| N0004 | (SNACKS) क्राइड पदार्थ | कच्चा चिवडा मुरमुरे | मुरमुरे;पाळीचे कांदे;कांदा;तेल;शेंगदाणे;दारल्या;पोहे |
| N0005 | (SNACKS) क्राइड पदार्थ | ब्रेड पकोडा | आलु;ब्रेड;बेसन |
| N0006 | (SNACKS) क्राइड पदार्थ | वडा पाव | आलु;मिर्ची;कांदा;बेसन;पाणी;तेल;पाव ब्रेड |
| N0007 | (SNACKS) क्राइड पदार्थ | ब्रेड भजे | ब्रेड;बेसन;कांदा |
| N0008 | (SNACKS) क्राइड पदार्थ | पोह्याचा चिवडा | पोहे;शेंगदाणे;दारल्या;कांदा;खोबरं;मिरची;तेल |
| N0009 | (SNACKS) क्राइड पदार्थ | मिरची भजे | मिरची;बेसन;तेल;पाणी |
| N0010 | (SNACKS) क्राइड पदार्थ | पोहे | जाडे पोहे;टमाटर;आलु;कांदा;तेल |
| N0011 | (SNACKS) क्राइड पदार्थ | शाबूदाणा उसळ | शाबूदाणा;दही;शेंगदाणे;तेल;आलु |
| N0012 | (SNACKS) क्राइड पदार्थ | शेंगदाणा भेल | शेंगदाणे |
| N0013 | (SNACKS) क्राइड पदार्थ | शिंगाडे शेव | शिंगाडे पीठ |
| N0014 | (SNACKS) क्राइड पदार्थ | प्याज पकोडा | कांदे;बेसन;पाणी;तेल |
| N0015 | (SNACKS) क्राइड पदार्थ | शिंगाडे पिठाचा पुऱ्या | शिंगाडे पीठ;तिखट |
| N0016 | (SNACKS) क्राइड पदार्थ | शिंगाडे पीठाचे आलु भजे | शिंगाडे पीठ;आलु |
| N0017 | (SNACKS) क्राइड पदार्थ | शाबूदाण वडा | शाबूदाणा;आलु;शेंगदाणे |

घरी बनविलेले गोड पदार्थ (Homemade sweets)

| **Code** | **food groups** | **Recipe name** | **Description of recipe** |
| --- | --- | --- | --- |
| T0001 | घरी बनविलेले गोड पदार्थ | तांदळाचे अनरसे | तांदुळ;साखर;तेल |
| T0002 | घरी बनविलेले गोड पदार्थ | गोड मलिंदा (गव्हाची पोळी) | कणिक;साखर;पाणी |
| T0003 | घरी बनविलेले गोड पदार्थ | साखरेची पोळी | साखर;कणिक;पाणी |
| T0004 | घरी बनविलेले गोड पदार्थ | रव्याची बर्फी | रवा;डालडा;खोबरं किस;पाणी;साखर; |
| T0005 | घरी बनविलेले गोड पदार्थ | तांदळाची खीर | तांदुळ;,साखर |
| T0006 | घरी बनविलेले गोड पदार्थ | तिळाचे मोदक | तीळ;गुळ;मैदा;पाणी |
| T0007 | घरी बनविलेले गोड पदार्थ | गोड भगर | भगर;पाणी;साखर;दुध |
| T0008 | घरी बनविलेले गोड पदार्थ | गुळाची पोळी | गुळ;शेंगदाणे;कणिक;पाणी;तीळ |
| T0009 | घरी बनविलेले गोड पदार्थ | गोड शंकरपाळे | मैदा;साखर;तेल |
| T0009 | घरी बनविलेले गोड पदार्थ | आलूचा शिरा | आलु;साखर;शेंगदाणे; |
| T0010 | घरी बनविलेले गोड पदार्थ | रव्याची खिर | रवा;साखर |
| T0011 | घरी बनविलेले गोड पदार्थ | साबुदाणा खिर | साबूदाना;साखर;पाणी;दुध |
| T0012 | घरी बनविलेले गोड पदार्थ | बुंदा | बेसन;साखर |
| T0013 | घरी बनविलेले गोड पदार्थ | खोबरा किसाचे लाडु | खोबर कीस;साखर;पाणी |
| T0014 | घरी बनविलेले गोड पदार्थ | कणकीचे बोंड | कणिक;साखर;पाणी;तेल |
| T0015 | घरी बनविलेले गोड पदार्थ | तिळाची करंजी | तीळ;गुळ;मैदा;तेल |
| T0016 | घरी बनविलेले गोड पदार्थ | मैद्याची दही पुरी | मैदा;दही;पाणी;साखर;तेल |
| T0017 | घरी बनविलेले गोड पदार्थ | पाक ब्रेड | ब्रेड;साखर;पाणी;तेल |
| T0018 | घरी बनविलेले गोड पदार्थ | तिळाचे लाडु | तीळ;गुळ;शेंगदाणे |
| T0019 | घरी बनविलेले गोड पदार्थ | तिळाची वडी | तीळ;साखर;शेंगदाणे |
| T0020 | घरी बनविलेले गोड पदार्थ | शिरा (प्याकेटचा शिरा ) | शिरयाचे प्याकेट,साखर;तेल;पाणी |
| T0021 | घरी बनविलेले गोड पदार्थ | आलुचे बोंड | आलु;कणिक;साखर;तेल |
| T0022 | घरी बनविलेले गोड पदार्थ | पाक पुरी | कणिक;साखर;पाणी;तेल |
| T0023 | घरी बनविलेले गोड पदार्थ | कोव्ल्याचे सुकुडे | कोवळ;साखर;कणिकपाणी;तेल |
| T0024 | घरी बनविलेले गोड पदार्थ | कोहळ्याच गुलशील | कोहळ;साखर;पाणी |
| T0025 | घरी बनविलेले गोड पदार्थ | राजगिरा लाडु | राजगिरा;गुळ |
| T0026 | घरी बनविलेले गोड पदार्थ | गोड भात | तांदुळ;साखर;पाणी |
| T0027 | घरी बनविलेले गोड पदार्थ | रव्याचा शिरा | रवा;डालडा;साखर;पाणी |
| T0028 | घरी बनविलेले गोड पदार्थ | तुरीचा गोड घुगऱ्या | तुरी;साखर |
| T0029 | घरी बनविलेले गोड पदार्थ | राजगिर्याची पुरी | राजगिरा पीठ;साखर;तेल |
| T0030 | घरी बनविलेले गोड पदार्थ | राज्गीरायाचा पीठाचे लाडू | राजगिरा पीठ;साखर;तूप;पाणी;शेंगदाणे |
| T0031 | घरी बनविलेले गोड पदार्थ | राजगिरा शिरा | राजगिरा;साखर;तूप;पाणी;शेंगदाणे |
| T0032 | घरी बनविलेले गोड पदार्थ | गोड खिचडा | चणाडाळ;गहू;ज्वारी;तांदुळ;साखर;पाणी |
| T0033 | घरी बनविलेले गोड पदार्थ | रस्को | मैदा;अंडे;साखर;तेल |
| T0034 | घरी बनविलेले गोड पदार्थ | चना गोड डाळ | चना;साखर;पाणी |
| T0035 | घरी बनविलेले गोड पदार्थ | कोवळ्याची खिर | कोवळ;साखर;दुध |
| T0036 | घरी बनविलेले गोड पदार्थ | दुध पुरी | शेंगदाणे; कणिक;साखर;तांदळाचपीठ;दुध;पाणी;तेल |
| T0037 | घरी बनविलेले गोड पदार्थ | केळीचे कालवण | केळी;साखर;दुध |
| T0038 | घरी बनविलेले गोड पदार्थ | उकळलेले बोर | बोर;साखर;पाणी |
| T0039 | घरी बनविलेले गोड पदार्थ | तीळ,गुळ पोळी | तीळ;गुळ;कणिक |
| T0040 | घरी बनविलेले गोड पदार्थ | रवा प्रसाद | रवा;डालडा;साखर |
| T0041 | घरी बनविलेले गोड पदार्थ | शेंगदाणे गुळ मोदक | शेंगदाणे;गुळ;पाणी |
| T0042 | घरी बनविलेले गोड पदार्थ | बुंदी रायता | बेसन;दही;साखर;कांदा |
| T0043 | घरी बनविलेले गोड पदार्थ | बीटचा हलवा गोड | बीट;साखर;तूप |
| T0044 | घरी बनविलेले गोड पदार्थ | पुरणाचे मोदक | चना डाळ;साखर |
| T0045 | घरी बनविलेले गोड पदार्थ | शेंगदाणा पापडी | शेंगदाणा;साखर |
| T0046 | घरी बनविलेले गोड पदार्थ | गव्हाचा सोजीचा ( संध्याची ) पोळी | सोजी(गहू);पाणी;साखर;डालडा;कणिक;तेल |
| T0047 | घरी बनविलेले गोड पदार्थ | बालुशाही | मैदा;डालडा;खोबर;दही;तेल;साखर |
| T0048 | घरी बनविलेले गोड पदार्थ | मुरमुर्याचे लाडू | मुरमुरे;गुळ;पाणी |
| T0049 | घरी बनविलेले गोड पदार्थ | पुरण पोळी | चना डाळ;साखर;कणिक;पाणी |
| T0050 | घरी बनविलेले गोड पदार्थ | शेंगदाणा लाडू | शेंगदाणे;गुळ;पाणी |
| T0051 | घरी बनविलेले गोड पदार्थ | गोड शेवल्या भाजलेल्या | शेवल्या;काजु;मनुका;दुध;साखर |
| T0052 | घरी बनविलेले गोड पदार्थ | गजर हलवा | गाजर;साखर;डालडाखोबरा किस;दुध |
| T0053 | घरी बनविलेले गोड पदार्थ | खिर | साखर;कणिक;पाणी;दुध;तूप |
| T0054 | घरी बनविलेले गोड पदार्थ | गुलाबजामुन | गुलाबजामुन पावडर;साखर;तेल;पाणी |
| T0055 | घरी बनविलेले गोड पदार्थ | रव्याचे लाडू | रवा;डालडा;साखर |
| T0056 | घरी बनविलेले गोड पदार्थ | रव्याचा शिरा | रवा;साखर;डालडा;काजु;दुध;पाणी |
| T0057 | घरी बनविलेले गोड पदार्थ | अप्पालू | तेल;साखर;कणिक;पाणी |
| T0058 | घरी बनविलेले गोड पदार्थ | कणकीचे लाडू | कणिक;डालडा;साखर;पाणी |
| T0059 | घरी बनविलेले गोड पदार्थ | मुंग दाल हलवा | मुंग दाल;खवा;तूप;साखर |
| T0060 | घरी बनविलेले गोड पदार्थ | पोह्याची खिर | पोहे;साखर;दुध;पाणी |
| T0061 | घरी बनविलेले गोड पदार्थ | काला जामून | खवा;साखर;मैदा;तेल;पाणी |
| T0062 | घरी बनविलेले गोड पदार्थ | कलरचे गुलाबजामून,चिरंजी | गुलाबजामून पावडर;साखर;तेल;पाणी;कलरची चिरंजी |
| T0063 | घरी बनविलेले गोड पदार्थ | शिंगाडा पिठाचा शिरा | शिंगाडा पीठ;साखर;तूप;पाणी |
| T0064 | घरी बनविलेले गोड पदार्थ | खव्याचे गुलाबजामून | खवा,साखर; |
| T0065 | घरी बनविलेले गोड पदार्थ | बेसन लाडू | बेसन;साखर;शेंगदाणे;तेल;खोबर;पाणी |
| T0066 | घरी बनविलेले गोड पदार्थ | रवा खिर | रवा;साखर |
| T0067 | घरी बनविलेले गोड पदार्थ | रतानाळ,दुध,साखर यांची खिर | रत्णाळ;दुध;साखर;पाणी; |
| T0068 | घरी बनविलेले गोड पदार्थ | रतनळाचे गोड बोंड | रतनाळे;साखर;पाणी;कणिक;तेल |
| T0069 | घरी बनविलेले गोड पदार्थ | THR शंकरपाळे | THR ;साखर;पाणी;तेल |
| T0070 | घरी बनविलेले गोड पदार्थ | THR लाडू | THR;साखर;पाणी |
| T0071 | घरी बनविलेले गोड पदार्थ | साखर आंबा | कच्चे आंबे;साखर; |
| T0072 | घरी बनविलेले गोड पदार्थ | रतनाळचा पुऱ्या | रतनाळ (कंद);कणिक;तेल;साखर |

**Drinks**

| **Code** | **food groups** | **Recipe name** | **Description of recipe** |
| --- | --- | --- | --- |
| Q0001 | चहा,कॉफी (HOT DRINK) | बकरीचा दुधाचा चहा | बकरीचे दुध;पाणी;साखर; |
| Q0002 | चहा,कॉफी (HOT DRINK) | गुळाचा चहा | गुळ;चहापत्ती |
| Q0003 | चहा,कॉफी (HOT DRINK) | कॉफी | दुध;साखर |
| Q0004 | चहा,कॉफी (HOT DRINK) | मसाला चाय | चहापत्ती;साखर;दुध |
| Q0005 | चहा,कॉफी (HOT DRINK) | लेमन टी | साखर;चहापत्ती;लिंबू;पाणी |
| Q0006 | चहा,कॉफी (HOT DRINK | म्हशीचा दुधाचा चहा | पाणी;साखर;दुध |
| Q0007 | चहा,कॉफी (HOT DRINK) | काला चहा | पाणी;चहापत्ती;साखर |
| Q0008 | चहा,कॉफी (HOT DRINK) | चहा | पाणी;साखर;दुध |
| Q0009 | चहा,कॉफी (HOT DRINK) | हळदीचा चहा | चहापत्ती;साखर |
| Q0009 | चहा,कॉफी (HOT DRINK) | हळदीचा चहा | पाणी;साखर;दुध |
| Q0010 | चहा,कॉफी (HOT DRINK) | मसाला चाय | दुध;साखर |
| R0001 | शरबत (COLD DRINK) | आंबाडी शरबत | आंबाडी फुल;साखर;पाणी |
| R0002 | COLD DRINK (ज्यूस) | मौसंभी ज्यूस | मौसंभी;साखर;पाणी |
| R0003 | COLD DRINK (ज्यूस) | BANANA शेक | केळी;साखर;दुध |
| R0004 | COLD DRINK (ज्यूस) | डांगर ज्यूस | डांगर;साखर;पाणी |
| R0005 | COLD DRINK (ज्यूस) | गाजर ज्यूस | गाजर;साखर;पाणी |
| R0006 | COLD DRINK (ज्यूस) | अंगूर ज्यूस | अंगूर;साखर |
| R0007 | COLD DRINK (ज्यूस) | अननस ज्यूस | अननस;साखर;पाणी |
| R0008 | COLD DRINK (ज्यूस) | टरबूज ज्यूस | टरबूज;साखर |
| R0009 | COLD DRINK (ज्यूस) | APPLE ज्यूस | APPLE;पाणी;साखर |
| R0010 | COLD DRINK (ज्यूस) | डाळिंबाचा ज्यूस | डाळिंब दाने;साखर;पाणी |
| R0011 | COLD DRINK (ज्यूस) | संत्री ज्यूस | संत्री;साखर |
| R0012 | COLD DRINK (ज्यूस) | लिंबू सरबत | लिंबू;पाणी;साखर |
| R0013 | COLD DRINK (ज्यूस) | आंब्याचे पन | आंबे;साखर |
| R0014 | COLD DRINK (ज्यूस) | आंब्याचे पन गोड | आंबे;साखर |

चटणी आणि लोणचे (Chutneys and Pickles)

| **Code** | **food groups** | **Recipe name** | **Description of recipe** |
| --- | --- | --- | --- |
| V0001 | चटणी आणि लोणचे | शेंगदाना,खोबऱ्याची चटणी | शेंगदाणे;खोबर; |
| V0002 | चटणी आणि लोणचे | पत्ताकोबी कच्ची चटनी | पत्ताकोबी;टमाटर;कांदा;दही |
| V0003 | चटणी आणि लोणचे | कच्चा कांद्याची चटणी | कांदे |
| V0004 | चटणी आणि लोणचे | खाकस,खोबऱ्याची;चटणी | खाकस;खोबर;टमाटर;कांदा |
| V0005 | चटणी आणि लोणचे | हिरव्या मिरचीच ठेचा (कच्चा) | हिरवी मिरची |
| V0006 | चटणी आणि लोणचे | चणाडाळ चटणी | चना डाळ;मिर्ची |
| V0007 | चटणी आणि लोणचे | कवटाची चटणी | कवट;मिर्ची;टमाटर |
| V0008 | चटणी आणि लोणचे | लिंबूचे लोणचं | लिंबू;साखर |
| V0009 | चटणी आणि लोणचे | मिरचीच लोणच | मिर्ची;गुळ |
| V0010 | चटणी आणि लोणचे | कांदा, टमाटर चटणी | मिर्ची;कांदा;टमाटरतेल |
| V0011 | चटणी आणि लोणचे | गाजराचे लोणचे | गाजर |
| V0012 | चटणी आणि लोणचे | कवठाची चटणी | कवट |
| V0013 | चटणी आणि लोणचे | लसून चटणी | लसून;हिरवी मिरची |
| V0014 | चटणी आणि लोणचे | तुरीचा दाण्याची चटणी | तुरीचे दाने |
| V0015 | चटणी आणि लोणचे | ओल्या लाल मिर्चीची चटणी | मिर्ची;टमाटर;कांदा |
| V0016 | चटणी आणि लोणचे | आवळ्याचे लोणचे | आवळे;साखर;तेल; |
| V0017 | चटणी आणि लोणचे | जवसाची चटणी | जवस;शेंगदाणे |
| V0018 | चटणी आणि लोणचे | तुरीचा दाळेची चटणी | तुर डाळ |
| V0019 | चटणी आणि लोणचे | आंबाडीचा फुलाची चटणी | आंबड फुल |
| V0020 | चटणी आणि लोणचे | इकोडीचे लोणचे | इकडोडी |
| V0021 | चटणी आणि लोणचे | कच्चा कांदा चटणी | कांदा |
| V0022 | चटणी आणि लोणचे | कांदा,टमाटर कच्ची चटणी | टमाटर,कांदा |
| V0023 | चटणी आणि लोणचे | काकडीची चटणी | काकडी;दही |
| V0024 | चटणी आणि लोणचे | शेंगदाणा चटणी | शेंगदाणे;मिर्ची;कांदा;टमाटर;तेल |
| V0025 | चटणी आणि लोणचे | मिरचीचा ठेचा (चटणी ) | मिरची;टमाटर |
| V0026 | चटणी आणि लोणचे | तीळ चटणी | तीळ;मिरची;तेल;कांदा;टमाटर |
| V0027 | चटणी आणि लोणचे | मुळ्याची चटणी | मुळा;मिरची;दही;तेल |
| V0028 | चटणी आणि लोणचे | टमाटरची चटणी | टमाटर;ओला कांदा;तेल |
| V0029 | चटणी आणि लोणचे | निंबू लोणच शिजवलेले | लिंबू;तेल |
| V0030 | चटणी आणि लोणचे | चना डाळ,खोबर चटणी | चना डाळ;ओले नारळ; |
| V0031 | चटणी आणि लोणचे | दालिया चटणी | दालिया;कांदा;हिरवी मिरची |
| V0032 | चटणी आणि लोणचे | कच्चा टमाटरचे लोणच | टमाटर;तेल |
| V0033 | चटणी आणि लोणचे | दह्याची चटणी | दही |
| V0034 | चटणी आणि लोणचे | लसून तडका चटणी | लसून |
| V0035 | चटणी आणि लोणचे | कच्चा दोडक्याच लोणच | दोडके |
| V0036 | चटणी आणि लोणचे | भाजलेल्या मिरची,टमातरची चटणी | मिरची;टमाटर |
| V0037 | चटणी आणि लोणचे | चवळी) दाण्याची चटणी | चवळी दाने |
| V0038 | चटणी आणि लोणचे | तिळाची चटणी भाजलेली | तीळ;हिरवी मिरची;पाणी |
| V0039 | चटणी आणि लोणचे | लाल मूल्याची चटणी | लाल मुळा;दही; |
| V0040 | चटणी आणि लोणचे | शेंगदाणा चटणी,दही मिरची | शेंगदाणे;मिरची;दही |
| V0041 | चटणी आणि लोणचे | आंब्याच लोणच | आंबा;तेल |
| V0042 | चटणी आणि लोणचे | चिंचेची चटणी | चिंच |
| V0043 | चटणी आणि लोणचे | आंबा,कांद्याची चटणी | आंबा |
| V0044 | चटणी आणि लोणचे | पदिनाची चटणी | पदिना पाने |
| V0045 | चटणी आणि लोणचे | ओल्या खोबऱ्याची चटणी | खोबर |
| V0046 | चटणी आणि लोणचे | कच्चा आंब्याची चटणी | कच्चा आंबा; |
| V0047 | चटणी आणि लोणचे | मेथी आंबा | आंबा;साखर;पाणी |
| V0048 | चटणी आणि लोणचे | गुळ आंबा | आंबा;गुलळ |
| V0049 | चटणी आणि लोणचे | कांद्यची चटणी | कांदे |

Fruits (Check the photo chart for different sizes)

| W001 | Fruits | अंगुर |
| --- | --- | --- |
| W002 | Fruits | काळे अंगूर |
| W003 | Fruits | टरबुज |
| W004 | Fruits | सफरचंद |
| W005 | Fruits | चिक्कू |
| W006 | Fruits | पपई |
| W007 | Fruits | आंबा (बदाम) |
| W008 | Fruits | डाळिंब |
| W009 | Fruits | संत्री |
| W0010 | Fruits | डांगर |
| W0011 | Fruits | मौसंभी |
| W0012 | Fruits | आंबा (हापूस ) |
| W0013 | Fruits | अननस |
| W0014 | Fruits | केळी |
| W0015 | Fruits | चीचबिली |
| W0016 | Fruits | कवट |

Raw Vegetables (Check the photo chart for different sizes)

| C0019 | पिवळा,छन्द्री भाजीपाला | गाजर |
| --- | --- | --- |
| C0020 | पिवळा,छन्द्री भाजीपाला | बीट |
| C0021 | पिवळा,छन्द्री भाजीपाला | रतनाळ |
| C0022 | पिवळा,छन्द्री भाजीपाला | काकडी |
| C0023 | पिवळा,छन्द्री भाजीपाला | कांदे (लाल ) |
| C0024 | पिवळा,छन्द्री भाजीपाला | कांदे (पांढरे) |
| C0025 | पिवळा,छन्द्री भाजीपाला | मुळा |
| C0026 | पिवळा,छन्द्री भाजीपाला | लिंबू |
| C0027 | पिवळा,छन्द्री भाजीपाला | बिनस |
| C0028 | पिवळा,छन्द्री भाजीपाला | भेर कोवळ |
| C0029 | पिवळा,छन्द्री भाजीपाला | काशी कोवळ |
| C0030 | पिवळा,छन्द्री भाजीपाला | कोवळ (शेंदरी) |
| C0031 | पिवळा,छन्द्री भाजीपाला | फणस |
| C0032 | पिवळा,छन्द्री भाजीपाला | सरकारी काकडी |
| C0033 | पिवळा,छन्द्री भाजीपाला | किरा काकडी |
| D0009 | मातीत उगवणारे | आलु |
| D0009 | मातीत उगवणारे | नवरगोल |
| E0045 | हिरवा भाजीपाला | कांदे (पालीचे) |
| E0046 | हिरवा भाजीपाला | मेथी |
| E0047 | हिरवा भाजीपाला | राजगिरा |
| E0048 | हिरवा भाजीपाला | लसणाचा पाला |
| F0098 | अन्य भाजीपाला | चपटा वाल |
| F0099 | अन्य भाजीपाला | साज वाल |
| F0100 | अन्य भाजीपाला | वाल दानेवाला |
| F0101 | अन्य भाजीपाला | पोपटी |
| F0102 | अन्य भाजीपाला | भेंडी |
| F0103 | अन्य भाजीपाला | लवकी |
| F0104 | अन्य भाजीपाला | चवळी शेंगा |
| F0105 | अन्य भाजीपाला | वांगे |
| F0106 | अन्य भाजीपाला | तेल दोडका |
| F0107 | अन्य भाजीपाला | पत्ता कोबी |
| F0108 | अन्य भाजीपाला | कारले |
| F0109 | अन्य भाजीपाला | टमाटर |
| F0110 | अन्य भाजीपाला | शिमला मिरची |
| F0111 | अन्य भाजीपाला | दोडके (पटचा ) |
| F0112 | अन्य भाजीपाला | फुलकोबी |
| F0113 | अन्य भाजीपाला | वांगे भरताचे |
| F0114 | अन्य भाजीपाला | वटाणा शेंगा |
| F0115 | अन्य भाजीपाला | लसन |
| F0116 | अन्य भाजीपाला | अद्रक |
| F0117 | अन्य भाजीपाला | मिरची |
| F0118 | अन्य भाजीपाला | तोंडरे |
| F0119 | अन्य भाजीपाला | मुंग्न्याचा शेंगा |
| F0120 | अन्य भाजीपाला | केली वांगे |
| F0121 | अन्य भाजीपाला | वाल (कथा ) |
| F0122 | अन्य भाजीपाला | बरबटीचा शेंगा |
| F0123 | अन्य भाजीपाला | भोबडी मिरची |
| F0124 | अन्य भाजीपाला | तुरीचा शेंगा |
| F0125 | अन्य भाजीपाला | वाळल्या मिरची |
| F0126 | अन्य भाजीपाला | नायलन शेंगा (लाल)3 |
| F0127 | अन्य भाजीपाला | कारले मसाला (लहान) |
| F0128 | अन्य भाजीपाला | केळी वांगे (जांभळे) |
| F0129 | अन्य भाजीपाला | परवल |
| F0130 | अन्य भाजीपाला | भूइमुंगाचा शेंगा |
| G0002 | कच्चे फळ(केळी,पपया) | आंबे(कच्चे) |
| G0003 | कच्चे फळ(केळी,पपया) | कच्चे केळ |
| N0018 | SNACK | मक्का कणीस |

Packaged foods

| **Code** | **Food group** | **Description** | **Recipe name (as it is in the packet)** |
| --- | --- | --- | --- |
| U001 | Packaged foods | SNEHA | सोयाबीन तेल |
| U002 | Packaged foods | DINSHAW'S | पनीर |
| U003 | Packaged foods | TOP LINE | मीठ |
| U004 | Packaged foods | SURUCHI | हळद |
| U005 | Packaged foods | DINSHAW'S | दुध |
| U006 | Packaged foods | NIRMA | मीठ |
| U007 | Packaged foods | MAGIC GOLD | गव्हाच पीठ |
| U008 | Packaged foods | RANI | बेसन (चना दाल) |
| U009 | Packaged foods | DECCAN | अद्रक पेस्ट |
| U010 | Packaged foods | RED TARGET | अद्रक व लसून पेस्ट |
| U011 | Packaged foods | KRISPY BITE | तोस्ट (ब्रेड) |
| U012 | Packaged foods | NEW FIVESTAR BAKERY | तोस्ट (ब्रेड) FRUIT |
| U013 | Packaged foods | GOLDEN | PREMIUM DARES |
| U014 | Packaged foods | PEPSI | खारी |
| U015 | Packaged foods | NANU | नांदेड चिवडा |
| U016 | Packaged foods | HALDIRAM'S | भूजीव शेव |
| U017 | Packaged foods | HALDIRAM'S | टका टक (कुरकुरे) |
| U018 | Packaged foods | DABANG | तोस्ट (ब्रेड) |
| U019 | Packaged foods | PARLE-G | भिस्कीट |
| U020 | Packaged foods | PARLE | क्रक्जाक भिस्कीट |
| U021 | Packaged foods | SURUCHI | गोडा मसाला |
| U022 | Packaged foods | HALDIRAM'S | सेव मुरमुरा |
| U023 | Packaged foods | HALDIRAM'S | मुंग दाल |
| U024 | Packaged foods | HALDIRAM'S | फलहारी चिवडा |
| U025 | Packaged foods | HALDIRAM'S | खट्टा मिठा (चिवडा) |
| U026 | Packaged foods | HALDIRAM'S | चिप्स |
| U027 | Packaged foods | BRITANNIA | BOURBON (भिस्कीट) |
| U028 | Packaged foods | PARLE | MONACO( भिस्कीट) |
| U029 | Packaged foods | PARLE | 20-20 (भिस्कीट) |
| U030 | Packaged foods | PARLE | MELODY (चॉकलेट) |
| U031 | Packaged foods | LAXMI NARAYAN | मिक्स नमकीन |
| U032 | Packaged foods | KRISHNA BRAYND R | पालमोलेन तेल |
| U033 | Packaged foods | SHREE | चिक्की |
| U034 | Packaged foods | NEW FIVESTAR BAKERY | मिल्क पाव |
| U035 | Packaged foods | NEW FIVESTAR BAKERY | तोस्ट मिठा 20-20 |
| U036 | Packaged foods | UPADHYAY'S | पापड |
| U037 | Packaged foods | SUNDAR | (कुरकुरे) CRUNCHY STICKS -TOMATO |
| U038 | Packaged foods | KRISHNA | TASTY SNACKS |
| U039 | Packaged foods | FUDAM | चिप्स |
| U040 | Packaged foods | VARSHANI'S | नमकीन |
| U041 | Packaged foods | PETULAL | NOODLES |
| U042 | Packaged foods | MAHARAJA | दबंग तोस्ट |
| U043 | Packaged foods | AMRUT | कुरकुरे |
| U044 | Packaged foods | KOFFEE | चॉकलेट |
| U045 | Packaged foods | GUAVA | चॉकलेट |
| U046 | Packaged foods | MANGO | चॉकलेट |
| U047 | Packaged foods | DARK CHOCO CUBE | चॉकलेट |
| U048 | Packaged foods | (KAMCO) PISTA | चॉकलेट |
| U049 | Packaged foods | STRAWBERRY | चॉकलेट |
| U050 | Packaged foods | KACHA AAM | चॉकलेट |
| U051 | Packaged foods | HALDIRAM'S | चिप्स (HALKE FULKE) |
| U052 | Packaged foods | VICKS | चॉकलेट |
| U053 | Packaged foods | ORIGINAL CARAMD | चॉकलेट |
| U054 | Packaged foods | DARK KISS | चॉकलेट |
